# Dengue on islands: a Bayesian approach to understanding the global ecology of dengue viruses

**DOI:** 10.1093/trstmh/trv012

**Published:** 2015-03-13

**Authors:** Leora R. Feldstein, John S. Brownstein, Oliver J. Brady, Simon I. Hay, Michael A. Johansson

**Affiliations:** a Children's Hospital Informatics Program, Boston Children's Hospital, 1 Autumn St., Boston, MA 02215, USA; b Center for Statistics and Quantitative Infectious Diseases, Vaccine and Infectious Disease Division, Fred Hutchinson Cancer Research Center, Seattle, Washington; USA; c Department of Pediatrics, Harvard Medical School, 1 Autumn St., Boston, MA 02215, USA; d Spatial Ecology and Epidemiology Group, Department of Zoology, University of Oxford, South Parks Road, Oxford, OX1 3PS, UK; e Fogarty International Center, National Institutes of Health, Bethesda, MD, USA; f Dengue Branch, Division of Vector-Borne Diseases, CDC, 1324 Calle Canada, San Juan, PR 00920, USA

**Keywords:** Dengue, Ecology, Epidemiology, Islands, Transmission dynamics

## Abstract

**Background:**

Transmission of dengue viruses (DENV), the most common arboviral pathogens globally, is influenced by many climatic and socioeconomic factors. However, the relative contributions of these factors on a global scale are unclear.

**Methods:**

We randomly selected 94 islands stratified by socioeconomic and geographic characteristics. With a Bayesian model, we assessed factors contributing to the probability of islands having a history of any dengue outbreaks and of having frequent outbreaks.

**Results:**

Minimum temperature was strongly associated with suitability for DENV transmission. Islands with a minimum monthly temperature of greater than 14.8**°**C (95% CI: 12.4–16.6**°**C) were predicted to be suitable for DENV transmission. Increased population size and precipitation were associated with increased outbreak frequency, but did not capture all of the variability. Predictions for 48 testing islands verified these findings.

**Conclusions:**

This analysis clarified two key components of DENV ecology: minimum temperature was the most important determinant of suitability; and endemicity was more likely in areas with high precipitation and large, but not necessarily dense, populations. Wealth and connectivity, in contrast, had no discernable effects. This model adds to our knowledge of global determinants of dengue risk and provides a basis for understanding the ecology of dengue endemicity.

## Introduction

Dengue viruses (DENV), vector-borne viruses of four distinct serotypes, have been estimated to cause as many as 280–530 million infections per year^1^ resulting in a range of manifestations from mild febrile illness to severe disease and death.^2,3^ Although DENV are widely distributed and are considered to be the most important arboviruses globally, there remains substantial uncertainty about transmission in some locations^4^ and about the most critical, population-level risk factors that contribute to either sporadic outbreaks or year-round transmission.

The dynamics and geography of DENV transmission have been extensively studied in a number of locations around the world. Since the early 1900s, it has been clear that DENV is transmitted by *Aedes aegypti* and *Ae. albopictus* mosquitoes. Populations of these species are limited by environmental conditions, such as temperature, precipitation and humidity.^5^ Their ability to transmit DENV is further dependent on temperature conditions which support DENV replication and dissemination within the mosquito,^6,7^ the survival of the adult female mosquito through that process^8^ and further feeding activity after becoming infectious.^9^ Socioeconomic factors are also important determinants of DENV transmission, particularly in relation to the frequency of contact between humans and mosquitoes. For instance, in areas with limited infrastructure, water storage practices^10–12^ and trash accumulation^13–17^ can provide aquatic habitats for immature mosquitoes in close proximity to homes. Meanwhile, the use of screens on windows and air conditioning can reduce mosquito–human interaction.^18^ Beyond a suitable environment for transmission, the occurrence of DENV transmission is dependent on the introduction of the virus which is spread by travelers, and a sufficiently large and dense susceptible human population, a factor somewhat complicated by the mix of short-term heterotypic and long-term homotypic immunity to DENV.^19–22^

While these climatic and socioeconomic factors are known to be important in some locations, their importance on a global scale remains unclear. Understanding their global importance is key to estimating dengue risk in places where little data is available and in projecting how dengue risk may change in the future as climate and socio-demographic conditions change. To identify factors that mediate DENV transmission risk and endemicity, we focused on DENV transmission on islands. Island populations are relatively isolated, such that transmission dynamics are more likely to be determined by local DENV ecology than by regional dynamics. Using a stratified random sample of inhabited islands throughout the world, we developed a database of dengue indicators from published literature, ministry of health data and informal digital sources. We then collected information on potentially relevant demographic, climate, socioeconomic, and connectivity variables to assess their relative contribution to the presence and endemicity of DENV in those island populations. This information along with data from the dengue database was incorporated into a Bayesian model with three components: the probability of observing a DENV outbreak if one occurred; the probability of climatic suitability for DENV transmission; and the probability of a DENV outbreak actually occurring. This model was fitted and then validated on a separate set of islands.

## Methods

### Island selection and covariate data

As a basis for selecting islands, we used a list of 1991 islands from the United Nations Environment Programme (UNEP; http://islands.unep.ch/isldir.htm). We collected the area of each island (km^2^) from the same database and extracted the latitude and longitude for each island by geocoding (www.spatialepidemiology.net). We then excluded all islands with area greater than 100 000 km^2^ and those located more than 45° from the equator, because DENV vector mosquitoes are unlikely to be found at more extreme latitudes.^5^ For the remaining 1319 islands, we obtained population size from the most recent national census databases. We then excluded all islands with an unknown population size or less than 100 inhabitants as islands with very sparse populations are numerous and unlikely to have reliable information on DENV transmission.

For the remaining 728 islands, we collected climate, economic, and connectivity data. We extracted monthly temperature, precipitation, and relative humidity estimates from the NOAA/NCEP Reanalysis (www.esrl.noaa.gov/psd/data/reanalysis).^23^ We calculated the average temperature, the average temperature for the coolest month of the year (minimum monthly temperature), average yearly precipitation, and average relative humidity over the past 20 years, 1993–2012. Country-level gross domestic product (GDP) per capita from 2011 (2012 US$) was collected from the United Nations Statistics Division (http://data.un.org/). As an indicator of connectivity, we obtained estimates of travel time from each island to cities ≥50 000 people (by plane or boat) from the European Commission Joint Research Centre Global Environment Monitoring Unit (http://bioval.jrc.ec.europa.eu/products/gam/download.htm).^24^

### Sampling strategy

We stratified the 728 islands by ocean (Atlantic, Indian, and Pacific), median GDP per capita (US$5318.13), and median population size (5354), creating a total of 12 categories. We randomly selected 12 islands from each of these 12 categories. We also selected another 12 islands more than 45° from the equator to capture characteristics associated with the limits of vector suitability. A total of 142 islands were included in the analysis (the low GDP and low population categories contained less than 12 islands). From each strata, we randomly selected two-thirds of islands for training and one-third of available islands for testing, resulting in 94 and 48 islands, respectively.

### Dengue data

We identified relevant published research using PubMed (http://www.ncbi.nlm.nih.gov/pubmed), and Google Scholar (http://www.scholar.google.com) and the search terms ‘dengue’ + island name, ‘dengue’ + country name, and ‘dengue’ + major city on the island (if applicable). We searched Ministry of Health and WHO reports by country using GIDEON (http://www.gideononline.com/) and Ministry of Health websites. We also identified a mix of formal and informal information about dengue outbreaks from ProMED (http://www.promedmail.org/), HealthMap (http://www.healthmap.org/), and Google (http://www.google.com).

We reviewed all the sources for evidence of dengue outbreaks on the selected islands. For the purpose of this study, we defined a local outbreak as any instance when at least 10 confirmed autochthonous cases were reported. For each island, we identified three principal indicators of local dengue transmission: if any outbreak had been recorded, the maximum number of consecutive years with dengue outbreaks with a maximum of ten, and the maximum number of years with outbreaks within a decade. For islands with no history of outbreaks, we also sought evidence of surveillance systems for influenza and vector-borne diseases. The presence of these systems was used an indicator of the likelihood of dengue being detected should it occur; in a location with no surveillance, detection is unlikely. The collected data and references are available in Supplementary Table 1.

### Statistical models

We estimated three different probabilities associated with the occurrence of dengue outbreaks: *p_Obs_*, the probability of observing an outbreak; *p_Suit_*, the probability of having a suitable climate for DENV transmission; and *p_Out_*, the yearly probability of having a dengue outbreak.

First, we modeled observation of at least one outbreak as a Bernoulli process dependent solely on *p_Obs_*:

$$O_{i}∼\mathrm{Bernoulli}(p_{Obs,i}),$$

where *O* indicates the presence or absence of at least one reported outbreak for each island (*i*), and *p_Obs_* is a logistic-linked linear function of the covariate *X*:

$$\text{logit}(p_{Obs,i})=\alpha_{0}+\alpha_{X}X_{i},$$

where *α_0_* is the intercept and *α_X_* is the coefficient for covariate *X* (e.g., population size). We fitted models using Bayesian Markov chain Monte Carlo sampling (described in detail below) and compared candidate models using the deviance information criteria (DIC)^25^.

Next, we fitted a model incorporating both *p_Obs_* and *p_Suit_* as determinants of the observation of at least one outbreak:

$$\begin{array}{ll} & O_{i}∼\mathrm{Bernoulli}(p_{Obs,i}p_{Suit,i}),\\ & \text{logit}(p_{Obs,i})\;=\;\alpha_{0}+\alpha_{X}X_{i},\\ & \text{logit}(p_{Suit,i})\;=\;\beta_{0}+\beta_{Y}Y_{i}, \end{array}$$

where the *X* covariates are those of the best *p_Obs_* model, the *α* coefficient priors are the posterior distributions from that model, *β_0_* is the intercept and *β_Y_* is the coefficient for covariate *Y* (e.g. average temperature). Using posteriors from the previous step for the *p_Obs_* component in the combined model helps isolate the expected independent effect of *p_Obs_*, but does not ensure that it remains significant. Using the *p_Obs_* components from the first stage we fitted alternative *p_Suit_* models, comparing the combined *p_Obs_* and *p_Suit_* models using DIC.

Finally, we defined the product, *p_Obs_p_Suit_p_Out,_* as the yearly probability of an outbreak occurring and being observed. We fitted models using two different observed outcomes. First, the number of consecutive years with dengue outbreaks (*C*) was assumed to come from a negative binomial distribution, i.e., how many years are likely to pass before there is a year without an outbreak:

$$C_{i}∼\mathrm{Negative}\;\;\mathrm{Binomial}(\text{1}-p_{Obs,i}p_{Suit,i}p_{Out,i},\text{1}).$$

Second, the maximum number of outbreak years within a decade (*D*) was assumed to come from a binomial distribution of the same yearly probability and the number of years, 10:

$$D_{i}∼\mathrm{Binomial}(\;p_{Obs,i}p_{Suit,i}p_{Out,i},\text{\textbackslash{}!1}0).$$

Again, each probability was treated as a logistic-linked linear function of the covariate:

$$\begin{array}{ll} & \text{logit}(p_{Obs,i})\;=\;\alpha_{0}+\alpha_{X}X_{i},\\ & \text{logit}(\;p_{Suit,i})\;=\;\beta_{0}+\beta_{Y}Y_{i},\text{and}\\ & \text{logit}(p_{Out,i})\;=\;\gamma_{0}+\gamma_{Z}Z_{i}, \end{array}$$

where the *X* and *Y* covariates are those of the best stage two model, the *α* and *β* coefficient priors are the posterior distributions from that model, *γ_0_* is the intercept and *γ_Z_* is the coefficient for covariate *Z* (e.g. population density). These two outcomes were fit simultaneously and the final model was selected by comparing the DIC of all candidate models.

We initially assigned weakly informative Gaussian priors to each *α*, *β*, and *γ* coefficient with zero means and variances based on the potential magnitude of their effects on the OR. For example, we expect a one-log change in population could change the OR by 0–50%. We thus set the prior SD to 0.5 such that 68% of the prior distribution fell between a 40% reduction and a 60% increase in the OR. The prior SD for the effects of the logged population density was also set to 0.5. For GDP and travel time, we used an SD of 0.1, equivalent to a 10% change per log change in GDP (in US$) or per log change in travel time (in minutes) to the nearest urban center, respectively. For temperature we used an SD of 0.2 for a 20% change per 1°C. Relative humidity and precipitation had greater ranges so we used an SD of 0.05, for 5% change per 1% change in relative humidity or per 1 cm of rain, respectively. Priors for the intercepts were set to zero with an SD of 2, such that there was strong prior coverage across the potential distributions of *p_Suit_*, *p_Obs_*, and *p_Out_*. We fitted each model in sequence using Bayesian Markov chain Monte Carlo sampling in OpenBugs 3.2.2 (http://www.openbugs.net),^26^ R2OpenBUGS (http://cran.r-project.org/web/packages/R2OpenBUGS/index.html)^27^ and R 3.0 (www.r-project.org). We mean-centered all covariates and thinned posterior samples every 10 samples to limit autocorrelation.

## Results

### Island data

Using a stratified sampling procedure, we selected 142 islands for training (94) and testing (48) datasets. These islands represented a variety of climates, demographics, and socioeconomic conditions (Table 1). We identified at least one dengue outbreak in 57 and 26 of the training and testing islands, respectively. For the remaining islands, we found no reported outbreaks, indicating either the absence of outbreaks or the absence of data documenting outbreaks. Of the 37 and 22 training and testing islands with no reported outbreaks, only 16 and 12, respectively, had evidence of local surveillance for vector-borne diseases or influenza, indicating the potential capacity to capture cases should they occur.

**Table 1.** Summary of data for training and testing islands

	Training set (n=94)		Testing set (n=48)	
Variable	Range	Median	Range	Median
Population size	177–23299716	6784	215–18943000	10683
Area (km^2^)	0.1–97724.1	178	0.9–84421	252.5
Population density (per km^2^)	0.2–9782.4	126.3	0.7–5147.8	80.4
Relative humidity (%)	29.6–93.4	80.6	64.1–92.3	80.8
Annual precipitation (cm)	3.3–426.9	154.2	7.4–426.9	172.6
Yearly average temperature (°C)	−9.1–29.1	26.2	5.5–27.6	26.3
Minimum monthly average temperature (°C)	−25.2–27.8	24.6	−6.8–27.0	24.8
GDP per capita (US$)	598–67039	7136.5	665–67039	8191.0
Travel time to nearest city (minutes)	4.0–5760.0	443.1	6.1–5760	379.5

### Model development

First, we analyzed the probability of having observed at least one outbreak on each island in the training dataset. We broke this probability down conceptually into two independent components: the probability of observing an outbreak should one occur; and the climatic suitability of the island for dengue transmission. Should a dengue outbreak occur, the probability of observing it (*p_Obs_*) may be reduced where there are very few people or few resources for surveillance. We therefore assessed the role of population size and socioeconomic status on observation. Assuming no difference in climatic suitability across the islands (which we address in the next step), we found that increased population size was associated with a greater probability of observing at least one outbreak, while GDP per capita had no association (Table 2).

We then added a climatic suitability component (*p_Suit_*) and assessed how suitability influences DENV transmission. Relative humidity and average annual precipitation had no association with suitability, but both average temperature and the minimum monthly temperature were positively associated, with the minimum monthly temperature model having a lower DIC value (Table 2). Therefore, the optimal model for observing at least one outbreak on an island included an observation component (*p_Obs_*) dependent on population size, and a suitability component (*p_Suit_*) dependent on the minimum monthly temperature.

**Table 2.** Odds ratios for univariate models

Component	Variable	OR	95% Credible Interval	DIC
*p*_*Obs*_	Population^a^	1.88	1.49–2.49	93
	GDP per capita (US$^a^)	0.95	0.80–1.14	128
*p*_*Suit*_	Precipitation (cm)	1.01	1.00–1.03	90
	Relative humidity (%)	1.06	0.96–1.14	88
	Average temperature (°C)	1.51	1.21–1.95	75
	Minimum temperature (°C)	1.51	1.24–1.91	68
*p*_*Out*_	Population^a^	1.38	1.27–1.50	677
	Travel time to nearest city (minutes^a^)	0.85	0.76–0.93	719
	Area (km^2a^)	1.26	1.17–1.38	705
	Population density (per km^2a^)	1.30	1.14–1.50	719
	GDP per capita (USD^a^)	1.16	1.01–1.33	720
	Precipitation (cm)	1.007	1.004–1.010	694
	Relative humidity (%)	1.08	1.04–1.13	709
	Average temperature (°C)	1.02	0.85–1.16	727
	Minimum temperature (°C)	1.04	0.90–1.15	726

DIC: deviance information criteria.

^a^ log-transformed.

Finally, we assessed factors in addition to those driving *p_Obs_* and *p_Suit_* that may contribute to the frequency of outbreaks, specifically the yearly probability of an outbreak occurring (*p_Out_*). As outcomes for this component, we used evidence of recurrent dengue outbreaks; the maximum number of years with outbreaks observed in a decade and the maximum number of consecutive years with outbreaks within a decade. The factors potentially influencing the frequency of outbreaks independent of the probability of observation and environmental suitability include demographics, connectivity, socioeconomics, and climate. Population size, population density, and area were all correlated and significantly associated with an increased yearly probability of outbreaks (Table 2). Increased travel time to the nearest city, i.e., decreased connectivity, was associated with decreased risk of outbreaks and higher GDP per capita was associated with increased risk of outbreaks. Of the climate variables, average annual precipitation and relative humidity were both associated with increased yearly probability of outbreaks. The model including population size was the best fitting model with a single covariate, as measured by the DIC (Table 2). Adding precipitation and relative humidity to the population model further reduced the DIC, while other additional covariates did not. The model with the lowest DIC included population and precipitation as covariates for the yearly probability of outbreaks occurring (Table 3).

**Table 3.** Coefficients for the best-fit model (DIC=650)

Component	Variable	Coefficient	95% Credible Interval
*p*_*Obs*_	Intercept	−5.3	−6.8, −3.8
	Population^a^	0.65	0.49, 0.81
*p*_*Suit*_	Intercept	−7.6	−10.8, −4.7
	Minimum temperature (°C)	0.51	0.36, 0.67
*p*_*Out*_	Intercept	−4.4	−5.5, −3.4
	Population^a^	0.31	0.24, 0.39
	Precipitation (cm)	0.0056	0.0035, 0.0078

DIC: deviance information criteria.

^a^ log-transformed.

**Figure 1.**
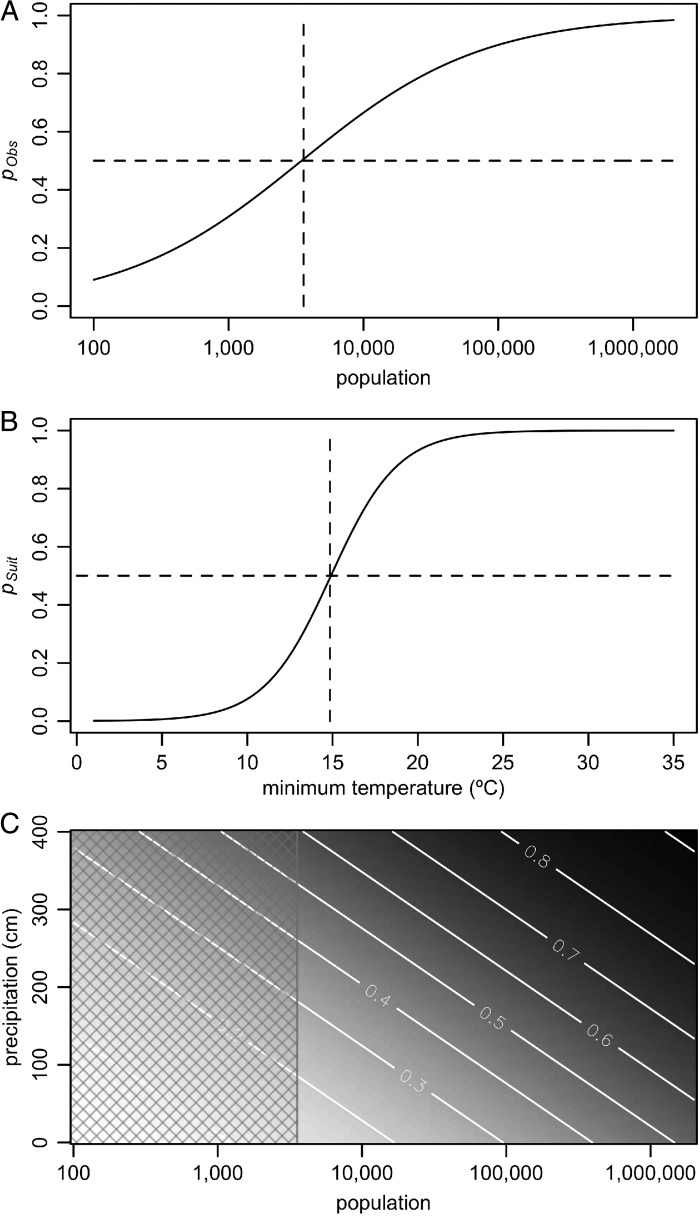
The effects of covariates on *p_Obs_*, *p_Suit_*, and *p_Out_*. (A) shows the probability of observing an outbreak at different population sizes. The threshold of *p_Obs_* = 0.5 is indicated with dashed lines. (B) shows the probability of an island being suitable for DENV transmission as minimum monthly temperature changes. The threshold of *p_Suit_* = 0.5 is indicated with dashed lines. (C) shows the relationship between population, precipitation, and the probability of recurrent outbreaks, with probability increasing from white to black. The contour lines indicate levels of *p_Out_* and the hatched area indicates the region where population size is too small for outbreaks to be readily observable (*p_Obs_* < 0.5)

The final model included three components: the probability of observing an outbreak, the probability of climatic suitability, and the yearly probability of outbreaks. This model included an effect of population on the probability of observation, an effect of minimum monthly temperature on the probability of suitability, and effects of population and precipitation on the yearly probability of outbreaks occurring. For every log increase in population size, the odds of observing an outbreak increased by 92% (95% credible interval [CI] 63–125%), with the probability of observation being greater than 0.5 for populations larger than 3600 (95% CI: 1900–5600) (Figure 1A). A 1**°**C increase in average temperature of the coldest month of the year was associated with a 67% (95% CI: 43–95%) higher odds of an island being suitable for DENV transmission. Islands with a minimum monthly temperature greater than 14.8**°**C (95% CI: 12.4–16.6**°**C) had a probability of suitability greater than 0.5 (Figure 1B). For every log increase in population size, the annual odds of an outbreak occurring increased by 36% (95% CI: 27–48%). For every centimeter increase in precipitation, the annual odds of an outbreak occurring increased by 0.56% (95% CI: 0.35–0.78%). Figure 1C shows the combined effect of these factors on the annual probability of outbreaks occurring.

### Model validation

**Figure 2.**
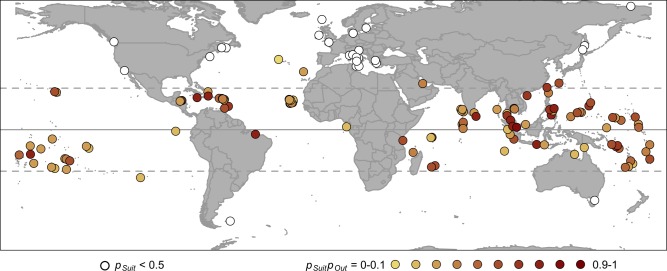
The global distribution of study islands. Each point represents an island from the study set. White points are islands with a low probability of suitability (*p_Suit_* <0.5). Colored islands are estimated to be suitable (*p_Suit_* >0.5) and to have varying probabilities of yearly outbreaks with *p_Suit_p_Out_* from 0–0.1 (yellow) to 0.9–1 (red). Credible intervals for these estimates are shown in Figure 3. The Equator is indicated by a solid line and the Tropics of Cancer and Capricorn, by dashed lines.

**Figure 3.**
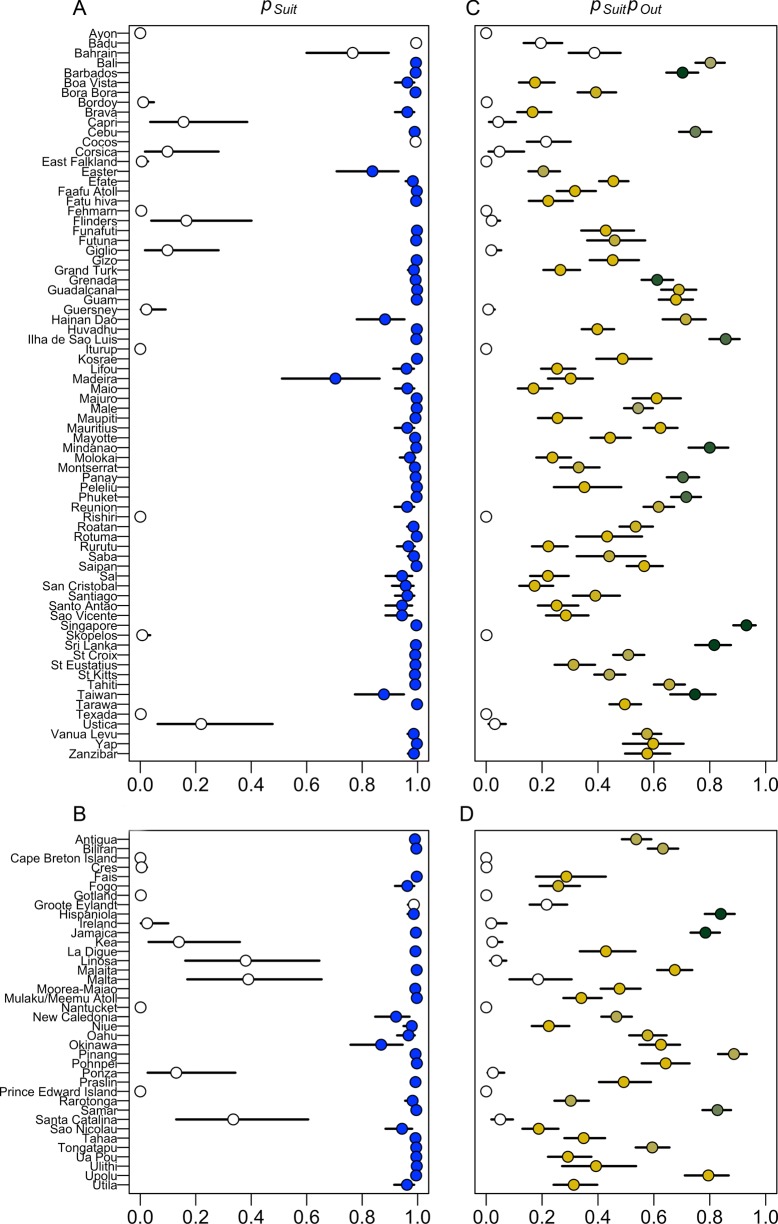
*p_Suit_* and *p_Suit_p_Out_* estimates for training and testing islands. (A) and (B) show the estimated *p_Suit_* (point) and 95% credible interval (line) for each island, in the training and testing datasets, respectively. White points are islands with no known history of dengue outbreaks, blue points are islands with evidence of at least one outbreak. (C) and (D) show the estimated *p_Suit_p_Out_* for each training and testing island, respectively. The colors indicate no outbreaks (white), and an increasing number of consecutive years with outbreaks from 1 (yellow) to 10 (dark green).

To assess the model's ability to identify suitable islands for dengue transmission we compared *p_Suit_* predictions to the data for both training and testing islands (Figures 2 and 3A). Of the 74 training islands with evidence of local surveillance or dengue outbreaks, the model predicted 60 to be suitable and 14 to be unsuitable. Three islands classified as suitable had no evidence of dengue transmission (Figure 3A). Two of these, Badu and Cocos, have small population sizes and thus are unlikely to have observed outbreaks (estimated *p_Obs_*= 0.27 and 0.23, respectively). The third island, Bahrain, was unique in the dataset as it had a large population and high temperatures, but the lowest humidity and precipitation among any of the islands. None of the islands classified as unsuitable had evidence of dengue outbreaks. The overall accuracy of *p_Suit_* for the training islands was 96% (57 of 60 islands predicted to be suitable had observed outbreaks and 14 of 14 islands predicted to be unsuitable had no evidence of transmission).

For the 38 testing islands with evidence of outbreaks or local surveillance, 11 had *p_Suit_* <0.5 and 27 had *p_Suit_* >0.5 (Figure 3B). Of the islands predicted to be unsuitable, none of them had evidence of dengue outbreaks. Of the 27 islands predicted to be suitable, only one had no evidence of an outbreak, Groote Eylandt, which is very small and had *p_Obs_* of 0.37. Overall the model classification for suitability was correct for 97% of the testing islands.

**Figure 4.**
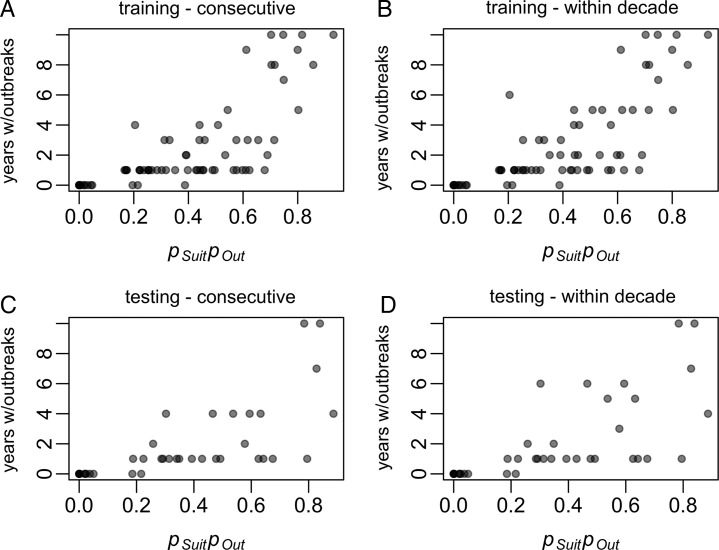
Model fit for *p_Suit_p_Out_* on training and testing data. (A) and (B) show the relationship between *p_Suit_p_Out_* estimates for the training dataset and the number of consecutive years with outbreaks and the maximum number of years with outbreaks within a decade, respectively. (R^2^=0.55 and 0.58, respectively). (C) and (D) show the relationship between *p_Suit_p_Out_* for the testing dataset and the number of consecutive years with outbreaks and the maximum number of years with outbreaks within a decade, respectively. (R^2^=0.49 and 0.47, respectively).

To assess the accuracy of the model to estimate endemicity, we multiplied *p_Suit_* and *p_Out_*, to consider locations that are suitable and likely to have frequent outbreaks. This index was strongly correlated with increased numbers of outbreaks in consecutive years and within a decade, accounting for 55 and 58% of the variability, respectively (Figure 4A–4B). While four training islands had long-term data indicative of endemicity (yearly outbreaks for a decade or more), 14 islands had less clear data, with 5–9 outbreak years in a decade. These islands and those with fewer outbreaks may be non-endemic areas with frequent outbreaks or areas with under-recognized transmission, a difference that is critical but difficult to assess. We thus evaluated the model accuracy against a somewhat arbitrary threshold, assuming that islands with at least five consecutive years of transmission were endemic and should have *p_Suit_p_Out_* >0.5. There were 49 training islands with *p_Suit_p_Out_* <0.5. Of these, all 49 had less than five consecutive years with outbreaks and 47 had less than five outbreak years in a decade (Figure 3C). Of the 25 training islands with *p_Suit_p_Out_* >0.5, 12 had at least five consecutive years with outbreaks and 16 had five or more outbreaks observed within a decade. The overall accuracy on the training data was 82% for consecutive years with outbreaks and 85% for outbreak years in a decade.

For the testing islands, *p_Suit_p_Out_* was also strongly associated with increased numbers of outbreaks in consecutive years and within a decade (accounting for 49% and 47% of the variability, respectively) (Figure 4C–4D). All of the 26 testing islands with *p_Suit_p_Out_* <0.5 had less than five consecutive years with outbreaks and less than five consecutive years with outbreaks. Of the 12 testing islands with *p_Suit_p_Out_* >0.5, three had at least five consecutive years with outbreaks and six had at least five outbreaks in a decade. The overall accuracy for the testing dataset was 76% for consecutive outbreak years and 79% for outbreaks in a decade.

## Discussion

Using demographic, socioeconomic, connectivity, climatic and dengue data from a stratified sample of 94 islands, we found that the occurrence of dengue outbreaks and their frequency is strongly associated with climate and population size. Minimum monthly temperature was the strongest determinant of climatic suitability, with locations predicted to be suitable having minimum monthly temperatures of approximately 14.8**°**C. Minimum temperature has often been found to have a strong association with both temporal^28–44^ and spatial^45,46^ variation in dengue incidence, highlighting the importance of low temperatures as a limiting factor of transmission. It has long been thought that the 10**°**C winter isotherm roughly defines the geographical limits of *Ae. aegypti* and DENV transmission.^5,47^ This threshold also seems appropriate given that *Ae. aegypti* infection with at least some viruses is possible at temperatures as low as 10**°**C.^48^ Nonetheless, *Ae. aegypti* activity is greatly reduced at these temperatures,^5^ so transmission, though possible, is generally unlikely. No islands in the training dataset with minimum monthly temperatures below 14.8**°**C had evidence of past dengue transmission, but other, colder, non-island areas clearly do (e.g., Philadelphia^49^ or Nice^50^). These areas are, however, much warmer during some parts of the year, so while areas with minimum temperatures below 14.8**°**C are unlikely to be hospitable for dengue transmission, there may be times when it can occur.

Precipitation and relative humidity had no discernible effects on climatic suitability across the islands. This may reflect adaptation of both humans and mosquitoes in areas or times of low rainfall where collected water often serves as larval habitat for *Ae. Aegypti*.^10,51–53^ Regardless of local climate, wherever there are humans, there is generally some sort of aquatic environment suitable for *Ae. aegypti* eggs, larvae, and pupae. Increased precipitation, however, was associated with an increased probability of outbreaks. Thus, although rainfall may not be critical for the occurrence of dengue, it may have a strong effect on the frequency of outbreaks.

Despite their biological importance to DENV transmission and the importance of temperature to climatic suitability, relative humidity and average temperature were not significantly associated with the yearly probability of outbreaks. It is still possible, however, that they have local effects in some locations. For example, low humidity may contribute to the absence of dengue outbreaks in Bahrain, the driest island in the dataset, suggesting that there may be a lower limit on humidity tolerance for DENV transmission that is not characterized here.

The most important determinant of the yearly outbreak probability in suitable environments was population size. Interestingly, population size had a stronger association than density. While high or moderate population density is a well-established risk factor for dengue,^18,54^ our finding suggests that on a population level, it may matter more how many people are functionally living in the same area rather than how closely they reside together. Indeed, increased spatial heterogeneity, which may be associated with slightly less dense populations, is a recognized contributor to pathogen persistence.^55–59^

After accounting for the effects of population and precipitation, country-level GDP per capita was not associated with differences in the probability of recurring outbreaks, with relatively wealthy islands like Singapore and relatively low-wealth islands, such as many of the Philippine islands, having evidence of frequent outbreaks. On the local level, it is likely that socioeconomics do influence dengue transmission, particularly related to water access, trash removal systems, the usage of screens or air conditioning, and housing density.^14,18,54,60,61^ Scale is likely a critical factor for these effects and even island-level effects may have been missed because of our reliance on country-level GDP data. Nonetheless, on the global scale, dengue does not appear to strictly respect economic boundaries.

Connectivity of islands to cities was associated with increased yearly risk of outbreaks in the univariate analysis. This implies an increased chance of frequent reintroduction or persistence of DENV transmission across regional meta-populations. However, proximity to cities is also related to population size, and connectivity had no significant association when population size was included in the model. Connectivity may certainly have a role in dengue dynamics for the islands considered here, but the related role of population size may have obscured it.

The final risk model included an effect of population size on observability, an effect of minimum temperature on climate suitability, and effects of population size and precipitation on outbreak frequency. Minimum temperature was an excellent predictor of whether outbreaks had ever occurred. Only four islands were predicted to be suitable despite a lack of evidence of outbreaks: three that were predicted to be too small to necessarily have evidence of outbreaks and a fourth, Bahrain, which represents a uniquely dry environment within this dataset where precipitation or humidity may play roles that are not evident elsewhere.

Accuracy was lower for prediction of outbreak frequency. Most of the discordance between the predicted and observed frequency of outbreaks occurred where the model predicted more frequent outbreaks than what was observed (Figure 4). This highlights three key limitations of this study. First, there may have been unrecognized or unreported outbreaks. Second, by design the model generalizes across all islands, thus cannot reflect heterogeneities that are not captured or factors beyond the scope of this study. Guam, Oahu, and Saipan, for example, suffered outbreaks during World War II, but have little or no evidence of more recent outbreaks.^62,63^ It is possible that vector control efforts, changing mosquito populations, or socioeconomic conditions have limited transmission to an extent that dengue is not endemic in these areas despite having conditions favoring endemicity.^62^ Lastly, the covariate data has its own limitations; at a global scale all must be estimated to some extent. The analysis could likely be improved by improving the quality of the covariate data.

While islands are not necessarily representative of mainland areas, they represent environments with unique characteristics that may be informative about the large-scale ecology of dengue for islands as well as non-island areas. Minimum temperature alone clearly differentiated suitable and unsuitable environments. Importantly, the data included few islands around the 14.8**°**C threshold, so the model may underestimate potential risk, especially in areas with strong seasonal variation that is generally reduced on islands. Future work can build on these results, investigating how dengue dynamics in mainland areas are related to these factors and others, such as seasonal weather variation^64^ and meta-population dynamics among closely connected locations.

Despite high accuracy in the model predictions, there are clearly other factors that influence DENV transmission, and it is not surprising that a simple global model did not capture all of the variation in the frequency of outbreaks. The areas where there is discordance between the model predictions and dengue data also provide direction for future research. In some areas with characteristics suggesting endemicity there may be unrecognized transmission, and in others, such as Guam, which has no evidence of transmission since 1944, further research may provide insight on how to control dengue.

### Conclusions

The key determinants identified here, minimum temperature, precipitation, and population size, have biological and empirical relationships with DENV transmission on a local scale. Although island ecology is not necessarily representative of the ecology of other, non-island locations, the relationships described here provide insight into the current global landscape of dengue and how it might change as climate and demographics change. Effective dengue prevention and control depends on understanding these factors and how they influence the spatiotemporal dynamics of dengue both now and in the future.

## Supplementary data

Supplementary data are available at Transactions Online (http://trstmh.oxfordjournals.org/).

Supplementary Data

## References

[1] Bhatt S Gething PW Brady OJ The global distribution and burden of dengue. Nature 2013;496:504–7. 23563266 10.1038/nature12060 PMC3651993

[2] WHO and Special Programme for Research and Training in Tropical Diseases. Dengue: Guideline for Diagnosis, Treatment, Prevention and Control. Geneva: World Health Organization; 2009.

[3] Simmons CP Farrar JJ Nguyenv V Dengue. N Engl J Med 2012;366:1423–32. 22494122 10.1056/NEJMra1110265

[4] Brady OJ Gething PW Bhatt S Refining the global spatial limits of dengue virus transmission by evidence-based consensus. PLoS Negl Trop Dis 2012;6:e1760. 22880140 10.1371/journal.pntd.0001760 PMC3413714

[5] Christophers SR *Aedes aegypti (L.)*: The Yellow Fever Mosquito. Cambridge: The University Press; 1960.

[6] Black WC Bennett KE Gorrochotegui-Escalante N Flavivirus susceptibility in *Aedes aegypti*. Arch Med Res 2002;33:379–88. 12234528 10.1016/s0188-4409(02)00373-9

[7] Chan M Johansson MA The incubation periods of dengue viruses. PLoS One 2012;7:e50972. 23226436 10.1371/journal.pone.0050972 PMC3511440

[8] Brady OJ Johansson MA Guerra CA Modelling adult *Aedes aegypti* and *Aedes albopictus* survival at different temperatures in laboratory and field settings. Parasit Vectors 2013;6:351. 24330720 10.1186/1756-3305-6-351 PMC3867219

[9] Pant CP Yasuno M Field studies on the gonotrophic cycle of *Aedes aegypti* in Bangkok, Thailand. J Med Entomol 1973;10:219–23. 4707760 10.1093/jmedent/10.2.219

[10] Ilkal MA Dhanda V Hassan MM Entomological investigations during outbreaks of dengue fever in certain villages in Maharashtra state. Indian J Med Res 1991;93:174–8. 1937596

[11] Barrera R Avila J Gonzalez-Tellez S Unreliable supply of potable water and elevated *Aedes aegypti* larval indices: a causal relationship? J Am Mosq Control Assoc 1993;9:189–95. 8350076

[12] Pontes RJ Freeman J Oliveira-Lima JW Vector densities that potentiate dengue outbreaks in a Brazilian city. Am J Trop Med Hyg 2000;62:378–83. 11037781 10.4269/ajtmh.2000.62.378

[13] Teng H-J Wu Y-L Lin T-H Mosquito fauna in water-holding containers with emphasis on dengue vectors (Diptera: Culicidae) in Chungho, Taipei County, Taiwan. J Med Entomol 1999;36:468–72. 10467775 10.1093/jmedent/36.4.468

[14] Thammapalo S Chongsuvivatwong V Geater A Environmental factors and incidence of dengue fever and dengue haemorrhagic fever in an urban area, Southern Thailand. Epidemiol Infect 2008;136:135–43. 17359563 10.1017/S0950268807008126 PMC2870760

[15] Almeida AS Medronho R de A Valencia LI Spatial analysis of dengue and the socioeconomic context of the city of Rio de Janeiro (Southeastern Brazil). Rev Saude Publica 2009;43:666–73. 19649472 10.1590/s0034-89102009000400013

[16] Arunachalam N Tana S Espino F Eco-bio-social determinants of dengue vector breeding: a multicountry study in urban and periurban Asia. Bull World Health Organ 2010;88:173–84. 20428384 10.2471/BLT.09.067892 PMC2828788

[17] Castro Peraza M Galvez Miranda C Sanchez Valdes L Community-based survey on knowledge and perceptions about dengue and preventive practice in Lisa municipality, City of Havana province [in Spanish]. Rev Cubana Med Trop 2010;62:245–53. 23437556

[18] Reiter P Lathrop S Bunning M Texas lifestyle limits transmission of dengue virus. Emerg Infect Dis 2003;9:86–9. 12533286 10.3201/eid0901.020220 PMC2873752

[19] Sabin AB Research on dengue during World War II. Am J Trop Med Hyg 1952;1:30–50. 14903434 10.4269/ajtmh.1952.1.30

[20] Gubler DJ Suharyono W Tan R Viraemia in patients with naturally acquired dengue infection. Bull World Health Organ 1981;59:623–30. 6976230 PMC2396101

[21] Gibbons RV Kalanarooj S Jarman RG Analysis of repeat hospital admissions for dengue to estimate the frequency of third or fourth dengue infections resulting in admissions and dengue hemorrhagic fever, and serotype sequences. Am J Trop Med Hyg 2007;77:910–3. 17984352

[22] Reich NG Shrestha S King AA Interactions between serotypes of dengue highlight epidemiological impact of cross-immunity. J R Soc Interface 2013;10:20130414. 23825116 10.1098/rsif.2013.0414 PMC3730691

[23] Kalnay E Kanamitsu M Kistler R The NCEP/NCAR 40-year reanalysis project. Bull Am Meteorol Soc 1996;77:437–71.

[24] Uchida H Nelson A WP/29 Agglomeration Index: Towards a New Measure of Urban Concentration. Helsinki: United Nations University-World Institute for Development Economics Research, 2010.

[25] Spiegelhalter DJ Best NG Carlin BR Bayesian measures of model complexity and fit. J R Stat Soc Series B Stat Methodol 2002;64:583–616.

[26] Lunn D Spiegelhalter D Thomas A The BUGS project: Evolution, critique and future directions. Stat Med 2009;28:3049–82. 19630097 10.1002/sim.3680

[27] Sturtz S Ligges U Gelman A R2WinBUGS: A package for running WinBUGS from R. J Stat Softw 2005;12:1–16.

[28] Nagao Y Thavara U Chitnumsup P Climatic and social risk factors for Aedes infestation in rural Thailand. Trop Med Int Health 2003;8:650–9. 12828549 10.1046/j.1365-3156.2003.01075.x

[29] Depradine C Lovell E Climatological variables and the incidence of dengue fever in Barbados. Int J Environ Health Res 2004;14:429–41. 15545038 10.1080/09603120400012868

[30] Promprou S Jaroensutasinee M Jaroensutasinee K Climatic factors affecting dengue haemorrhagic fever incidence in southern Thailand. Dengue Bull 2005;29:41.

[31] Chowell G Sanchez F Climate-based descriptive models of dengue fever: the 2002 epidemic in Colima, Mexico. J Environ Health 2006;68:40–4. 16780000

[32] Hurtado-Díaz M Riojas-Rodríguez H Rothenberg SJ Impact of climate variability on the incidence of dengue in Mexico. Trop Med Int Health 2007;12:1327–37. 17956543 10.1111/j.1365-3156.2007.01930.x

[33] Chowell G Torre CA Munayco-Escate C Spatial and temporal dynamics of dengue fever in Peru: 1994–2006. Epidemiol Infect 2008;136:1667–77. 18394264 10.1017/S0950268808000290 PMC2870787

[34] Lu L Lin H Tian L Time series analysis of dengue fever and weather in Guangzhou, China. BMC Public Health 2009;9:395. 19860867 10.1186/1471-2458-9-395 PMC2771015

[35] Chen SC Liao CM Chio CP Lagged temperature effect with mosquito transmission potential explains dengue variability in southern Taiwan: insights from a statistical analysis. Sci Total Environ 2010;408:4069–75. 20542536 10.1016/j.scitotenv.2010.05.021

[36] Colon-Gonzalez FJ Lake IR Bentham G Climate variability and dengue fever in warm and humid Mexico. Am J Trop Med Hyg 2011;84:757–63. 21540386 10.4269/ajtmh.2011.10-0609 PMC3083744

[37] Estallo EL Luduena-Almeida FF Visintin AM Prevention of dengue outbreaks through *Aedes aegypti* oviposition activity forecasting method. Vector Borne Zoonotic Dis 2011;11:543–9. 20925528 10.1089/vbz.2009.0165

[38] Gharbi M Quenel P Gustave J Time series analysis of dengue incidence in Guadeloupe, French West Indies: forecasting models using climate variables as predictors. BMC Infect Dis 2011;11:166. 21658238 10.1186/1471-2334-11-166 PMC3128053

[39] Pinto E Coelho M Oliver L The influence of climate variables on dengue in Singapore. Int J Environ Health Res 2011;21:415–26. 21557124 10.1080/09603123.2011.572279

[40] Yu H-L Yang S-J Yen H-J A spatio-temporal climate-based model of early dengue fever warning in southern Taiwan. Stoch Env Res Risk A 2011;25:485–94.

[41] Gomes AF Nobre AA Cruz OG; Temporal analysis of the relationship between dengue and meteorological variables in the city of Rio de Janeiro, Brazil, 2001–2009. Cad Saude Publica 2012;28:2189–97. 23147960 10.1590/s0102-311x2012001100018

[42] Simoes TC Codeco CT Nobre AA Modeling the non-stationary climate dependent temporal dynamics of *Aedes aegypti*. PLoS One 2013;8:e64773. 23976939 10.1371/journal.pone.0064773 PMC3748059

[43] Wang C Jiang B Fan J A study of the dengue epidemic and meteorological factors in Guangzhou, China, by using a zero-inflated poisson regression model. Asia Pac J Public Health 2014:26:48–57. 23761588 10.1177/1010539513490195

[44] Lourenco J Recker M The 2012 Madeira dengue outbreak: epidemiological determinants and future epidemic potential. PLoS Negl Trop Dis 2014;8:e3083. 25144749 10.1371/journal.pntd.0003083 PMC4140668

[45] de Garin AB Bejaran RA Carbajo AE Atmospheric control of *Aedes aegypti* populations in Buenos Aires (Argentina) and its variability. Int J Biometeorol 2000;44:148–56. 11049004 10.1007/s004840000051

[46] Barcellos C Lowe R Expansion of the dengue transmission area in Brazil: the role of climate and cities. Trop Med Int Health 2014;19:159–68. 24286460 10.1111/tmi.12227

[47] WHO. Dengue haemorrhagic fever: diagnosis, treatment, prevention and control. 2nd ed Geneva: World Health Organization; 1997.

[48] Turell MJ Lundstrom JO Effect of environmental temperature on the vector competence of *Aedes aegypti* and *Ae**.* *taeniorhynchus* for Ockelbo virus. Am J Trop Med Hyg 1990;43:543–50. 10.4269/ajtmh.1990.43.543 2173434

[49] Rush AB An Account of the Bilious Remitting Fever, as it Appeared in Philadelphia in the Summer and Autumn of the Year 1780. Medical Inquiries and Observations. Philadelphia: Prichard and Hall; 1789 p. 104–117.

[50] La Ruche G Souares Y Armengaud A First two autochthonous dengue virus infections in metropolitan France, September 2010. Euro Surveill 2010;15:19676. 20929659

[51] Gagnon AS Bush ABG Smoyer-Tomic KE Dengue epidemics and the El Niño Southern Oscillation. Clim Res 2001;19:35–43.

[52] Padmanabha H Soto E Mosquera M Ecological links between water storage behaviors and *Aedes aegypti* production: implications for dengue vector control in variable climates. Ecohealth 2010;7:78–90. 20358255 10.1007/s10393-010-0301-6

[53] Seidahmed OM Hassan SA Soghaier MA Spatial and temporal patterns of dengue transmission along a Red Sea coastline: a longitudinal entomological and serological survey in Port Sudan city. PLoS Negl Trop Dis 2012;6:e1821. 23029582 10.1371/journal.pntd.0001821 PMC3459851

[54] Schmidt WP Suzuki M Thiem VD Population density, water supply, and the risk of dengue fever in Vietnam: cohort study and spatial analysis. PLoS Med 2011;8:e1001082. 21918642 10.1371/journal.pmed.1001082 PMC3168879

[55] Grenfell B Harwood J (Meta)population dynamics of infectious diseases. Trends Ecol Evol 1997;12:395–9. 21238122 10.1016/s0169-5347(97)01174-9

[56] Keeling MJ Rohani P Estimating spatial coupling in epidemiological systems: a mechanistic approach. Ecol Lett 2002;5:20–9.

[57] Hagenaars TJ Donnelly CA Ferguson NM Spatial heterogeneity and the persistence of infectious diseases. J Theor Biol 2004;229:349–59. 15234202 10.1016/j.jtbi.2004.04.002

[58] Read JM Keeling MJ Disease evolution on networks: the role of contact structure. Proc Biol Sci 2003;270:699–708. 12713743 10.1098/rspb.2002.2305 PMC1691304

[59] Lourenco J Recker M Natural, persistent oscillations in a spatial multi-strain disease system with application to dengue. PLoS Comput Biol 2013;9:e1003308. 24204241 10.1371/journal.pcbi.1003308 PMC3812071

[60] Gubler DJ Dengue and dengue hemorrhagic fever: its history and resurgence as a global public health problem. In: Gubler DJ Kuno Gs (editors). Dengue and Dengue Hemorrhagic Fever. Wallingford: CABI; 1997 p. 1–22.

[61] Kuno G Factors influencing the transmission of dengue viruses. In: GublerKuno DJGs (editors). Dengue and Dengue Hemorrhagic Fever. Wallingford: CABI; 1997 p. 61–88.

[62] Lambrechts L Scott TW Gubler DJ Consequences of the expanding global distribution of *Aedes albopictus* for dengue virus transmission. PLoS Negl Trop Dis 2010;4:e646. 20520794 10.1371/journal.pntd.0000646 PMC2876112

[63] Gibbons RV Streitz M Babina T Dengue and US military operations from the Spanish-American War through today. Emerg Infect Dis 2012;18:623–30. 22469290 10.3201/eid1804.110134 PMC3309667

[64] Brady OJ Golding N Pigott DM Global temperature constraints on *Aedes aegypti* and *Ae**.* *albopictus* persistence and competence for dengue virus transmission. Parasit Vectors 2014;7:338. 10.1186/1756-3305-7-338 PMC4148136 25052008

