# Supplementary material for: Dengue on islands: a Bayesian approach to understanding the global ecology of dengue viruses
Source: Trans R Soc Trop Med Hyg. 2015 Mar 13;109(5):303–12. doi: 10.1093/trstmh/trv012 (PMC4401210; doi:10.1093/trstmh/trv012)
Supplement: Supplementary Data [file supp_109_5_303__index.html]

Dengue on islands: a Bayesian approach to understanding the global ecology of dengue viruses — Dengue on islands: a Bayesian approach to understanding the global ecology of dengue viruses — Supplementary Data 

# Dengue on islands: a Bayesian approach to understanding the global ecology of dengue viruses

## Supplementary Data

Supplementary Data

**Files in this Data Supplement:**

- Supplementary Data - Docx file
