# Supplementary material for: Dengue on islands: a Bayesian approach to understanding the global ecology of dengue viruses
Source: Trans R Soc Trop Med Hyg. 2015 Mar 13;109(5):303–12. doi: 10.1093/trstmh/trv012 (PMC4401210; doi:10.1093/trstmh/trv012)
Supplement: Supplementary Data [file supp_trv012_trv012supp.docx]

**Supplementary Table 1.** Island-specific dengue data

| Island | Country | Years with reported outbreaks | Reference | Maximum number of consecutive outbreak years | Maximum number of years with outbreaks in a decade | Island–specific disease surveillance |
| --- | --- | --- | --- | --- | --- | --- |
| Aniwa | Vanuatu |  | NA | 0 | 0 | None found |
| Antigua | Antigua and Barbuda | 1828, 1964, 1968, 1981, 1995–1998, 2001, 2012 | 1–4 | 4 | 5 | ND |
| Ayon | Russia | None | NA | 0 | 0 | None found |
| Badu | Australia | None | NA | 0 | 0 | [Australian DoH](http://www9.health.gov.au/cda/source/rpt_1.cfm?RequestTimeout=500) |
| Bahrain | Bahrain | None | NA | 0 | 0 | [MoH](http://www.moh.gov.bh/PDF/Publications/statistics/HS2012/hs2012_e.htm) |
| Bali | Indonesia | 1990–1993, 2006–2010 | 5–10 | 5 | 5 | ND |
| Barbados | Barbados | 1977, 1982–1984, 1987–1998, 2001–2012 | 2, 4, 11–13 | 12 | 10 | ND |
| Biliran | Philippines | 2008–2011, 2013 | 14–18 | 4 | 5 | ND |
| Bitra | India | None | NA | 0 | 0 | None found |
| Boa Vista | Cape Verde | 2009 | 19 | 1 | 1 | ND |
| Bora Bora | French Polynesia | 1996, 2001, 2008–2009 | 20–23 | 2 | 3 | ND |
| Bordoy | Denmark | None | NA | 0 | 0 | [European CDC](http://www.ecdc.europa.eu/) |
| Brava | Cape Verde | 2009 | 24 | 1 | 1 | ND |
| Butaritari | Kiribati | None | NA | 0 | 0 | None found |
| Cape Breton Island | Canada | None | NA | 0 | 0 | [PHA of Canada](http://www.phac-aspc.gc.ca/fluwatch/) |
| Capri | Italy | None | NA | 0 | 0 | [European CDC](http://www.ecdc.europa.eu/) |
| Cebu | Philippines | 1982–1988, 1999, 2009–2013 | 25–29 | 7 | 7 | ND |
| Cocos | Australia | None | NA | 0 | 0 | [Australian DoH](http://www9.health.gov.au/cda/source/rpt_1.cfm?RequestTimeout=500) |
| Corsica | France | None | NA | 0 | 0 | [European CDC](http://www.ecdc.europa.eu/) |
| Corvo | Portugal | None | NA | 0 | 0 | None found |
| Cres | Croatia | None | NA | 0 | 0 | [European CDC](http://www.ecdc.europa.eu/) |
| Delft | Sri Lanka | None | NA | 0 | 0 | None found |
| East Falkland | The Falklands | None | NA | 0 | 0 | [Eurosurveillance report](http://www.eurosurveillance.org/ViewArticle.aspx?ArticleId=19923) |
| Easter | Chile | 2000, 2002, 2006–2009, 2011 | 30–32 | 4 | 6 | ND |
| Efate | Vanuatu | 1998, 2008, 2010 | 33–35 | 1 | 2 | ND |
| Enggano | Indonesia | None | NA | 0 | 0 | None found |
| Faafu Atoll | Maldives | 2011 | 36 | 1 | 1 | ND |
| Fais | Federated States of Micronesia | 2012 | 37 | 1 | 1 | ND |
| Fatu hiva | French Polynesia | 2001 | 38 | 1 | 1 | ND |
| Fehmarn | Germany | None | NA | 0 | 0 | [European CDC](http://www.ecdc.europa.eu/) |
| Felidu | Maldives | None | NA | 0 | 0 | None found |
| Flinders | Australia | None | NA | 0 | 0 | [Australian DoH](http://www9.health.gov.au/cda/source/rpt_1.cfm?RequestTimeout=500) |
| Fogo | Cape Verde | 2009–2010 | 39–41 | 2 | 2 | ND |
| Funafuti | Tuvalu | 1945, 1989 | 42–43 | 1 | 1 | ND |
| Futuna | France | 1971–1972, 1976–1977, 1989, 2002–2004 | 44–48 | 3 | 4 | ND |
| Giglio | Italy | None | NA | 0 | 0 | [European CDC](http://www.ecdc.europa.eu/) |
| Gizo | Solomon Islands | 2013 | 49 | 1 | 1 | ND |
| Gotland | Sweden | None | NA | 0 | 0 | [European CDC](http://www.ecdc.europa.eu/) |
| Grand Turk | United Kingdom | 1974, 2012 | 4, 50 | 1 | 1 | ND |
| Grenada | Grenada | 1969–1971, 1989, 1995–2003, 2008–2012 | 4, 12, 13, 21, 51–53 | 9 | 9 | ND |
| Groote Eylandt | Australia | None | NA | 0 | 0 | [Australian DoH](http://www9.health.gov.au/cda/source/rpt_1.cfm?RequestTimeout=500) |
| Guadalcanal | Solomon Islands | 1982, 1995–1996, 2013 | 54–55 | 2 | 2 | ND |
| Guam | United States | 1944 | 56 | 1 | 1 | ND |
| Guanaja | Honduras | None | NA | 0 | 0 | None found |
| Guersney | United Kingdom | None | NA | 0 | 0 | [European CDC](http://www.ecdc.europa.eu/) |
| Hainan Dao | People's Republic of China | 1979–1982, 1985–1986, 1991 | 57–61 | 3 | 5 | ND |
| Hispaniola | Haiti/ Dominican Republic | 1960–1965, 1969, 1978–2013 | 3, 62–68 | 36 | 10 | ND |
| Huvadhu | Maldives | 2011 | 69 | 1 | 1 | ND |
| Ilha de Sao Luis | Brazil | 1995–2002 | 70–72 | 8 | 8 | ND |
| Ireland | Ireland | None | NA | 0 | 0 | [European CDC](http://www.ecdc.europa.eu/) |
| Itbayat | Philippines | None | NA | 0 | 0 | None found |
| Iturup | Russia | None | NA | 0 | 0 | None found |
| Jamaica | Jamaica | 1963–1975, 1995, 1998, 2001–2012 | 12, 13, 53, 73–80 | 13 | 10 | ND |
| Kalpeni | India | None | NA | 0 | 0 | None found |
| Kapingamarangi | Federated States of Micronesia | None | NA | 0 | 0 | None found |
| Kavaratti | India | None | NA | 0 | 0 | None found |
| Kea | Greece | None | NA | 0 | 0 | [European CDC](http://www.ecdc.europa.eu/) |
| Kiltan | India | None | NA | 0 | 0 | None found |
| Ko Chang | Thailand | None | NA | 0 | 0 | None found |
| Ko Kut | Thailand | None | NA | 0 | 0 | None found |
| Ko Phra Thong | Thailand | None | NA | 0 | 0 | None found |
| Komodo | Indonesia | None | NA | 0 | 0 | None found |
| Kosrae | Federated States of Micronesia | 1998 | 81 | 1 | 1 | ND |
| La Digue | Seychelles | 1977, 2013 | 82–83 | 1 | 1 | ND |
| Lifou | New Caledonia | 1983, 2004, 2008, 2013 | 84–87 | 1 | 3 | ND |
| Linosa | Italy | None | NA | 0 | 0 | [European CDC](http://www.ecdc.europa.eu/) |
| Lord Howe Atoll | Solomon Islands | None | NA | 0 | 0 | None found |
| Madeira | Portugal | 2012 | 74, 88–89 | 1 | 1 | ND |
| Maio | Cape Verde | 2009 | 24 | 1 | 1 | ND |
| Majuro | Marshall Islands | 2003, 2011 | 90–91 | 1 | 2 | ND |
| Malaita | Solomon Islands | 2013 | 92 | 1 | 1 | ND |
| Male | Maldives | 1988, 1998–1999, 2008–2012 | 93–99 | 5 | 5 | ND |
| Malta | Malta | None | NA | 0 | 0 | [European CDC](http://www.ecdc.europa.eu/) |
| Mangaia | Cook Islands | None | NA | 0 | 0 | None found |
| Maupiti | French Polynesia | 2009 | 100 | 1 | 1 | ND |
| Mauritius | Mauritius | 2009 | 101 | 1 | 1 | ND |
| Mayotte | France | 1943, 2010, 2012 | 102–104 | 1 | 2 | ND |
| Mindanao | Philippines | 2005–2013 | 100, 105–108 | 9 | 9 | ND |
| Mokil | Federated States of Micronesia | None | NA | 0 | 0 | None found |
| Molokai | United States | 1944 | 108 | 1 | 1 | ND |
| Montserrat | United Kingdom | 1994–1996, 2002–2003, 2011 | 109–112 | 3 | 3 | ND |
| Moorea–Maiao | French Polynesia | 1979, 2001, 2013 | 21, 113–114 | 1 | 1 | ND |
| Mulaku/Meemu Atoll | Maldives | 2011 | 115 | 1 | 1 | ND |
| Nantucket | United States | None | NA | 0 | 0 | [CDC](http://www.cdc.gov/flu/weekly) |
| New Caledonia | New Caledonia | 1976–1977, 1995–1998, 2002–2004, 2007–2009 | 116 | 4 | 6 | ND |
| Niue | Niue | 1972, 1980, 1986 | 117 | 1 | 1 | ND |
| Nukunonu | New Zealand | 2001 | 118 | 0 | 0 | None found |
| Oahu | United States | 1944, 2001–2002, 2011 | 108, 119 | 2 | 3 | ND |
| Okinawa | Japan | 1904, 1930, 1945 | 120, 121 | 1 | 1 | ND |
| Pacijan | Philippines | None | NA | 0 | 0 | None found |
| Pagai Utara | Indonesia | None | NA | 0 | 0 | None found |
| Panay | Philippines | 2006–2013 | 122–126 | 8 | 8 | ND |
| Peleliu | Palau | 1988, 1995 | 127 | 1 | 2 | ND |
| Phuket | Thailand | 2006–2013 | 128–132 | 8 | 8 | ND |
| Pinang | Malaysia | 1962, 1974, 1978, 1982, 1990, 1995, 2009–2012 | 133–138 | 4 | 4 | ND |
| Pini | Indonesia | None | NA | 0 | 0 | None found |
| Pohnpei | Federated States of Micronesia | 1992 | 139 | 1 | 1 | ND |
| Ponza | Italy | None | NA | 0 | 0 | [European CDC](http://www.ecdc.europa.eu/) |
| Praslin | Seychelles | 1977 | 82 | 1 | 1 | ND |
| Prince Edward Island | Canada | None | NA | 0 | 0 | [PHA of Canada](http://www.phac-aspc.gc.ca/fluwatch/) |
| Principe | Sao Tome and Principe | None | NA | 0 | 0 | None found |
| Pukapuka | Cook Islands | None | NA | 0 | 0 | None found |
| Rarotonga | Cook Islands | 1991, 1995, 1997, 2001–2002, 2006–2009 | 140–141 | 4 | 6 | ND |
| Reunion | France | 1977, 1978, 2004, 2007–2009, 2012 | 142–143 | 3 | 5 | ND |
| Rishiri | Japan | None | NA | 0 | 0 | [IDSC](http://idsc.nih.go.jp/) |
| Roatan | Honduras | 1978–1979 | 144 | 2 | 2 | ND |
| Rotuma | Fiji | 1972 | 145 | 1 | 1 | ND |
| Rupat | Indonesia | None | NA | 0 | 0 | None found |
| Rurutu | French Polynesia | 2009 | 100 | 1 | 1 | ND |
| Rutland | India | None | NA | 0 | 0 | None found |
| Saba | Netherlands | 2008, 2010–2012 | 4, 146–147 | 3 | 4 | ND |
| Saipan | United States | 1944, 1971 | 121, 148 | 1 | 1 | ND |
| Sal | Cape Verde | 2009 | 149 | 1 | 1 | ND |
| Samar | Philippines | 2007–2013 | 150–157 | 7 | 7 | ND |
| San Cristobal | Ecuador | 2010 | 158 | 1 | 1 | ND |
| Santa Catalina | United States | None | NA | 0 | 0 | [CDC](http://www.cdc.gov/flu/weekly) |
| Santiago | Cape Verde | 2009–2010 | 24, 39, 159 | 2 | 2 | ND |
| Santo Antao | Cape Verde | 2009 | 19 | 1 | 1 | ND |
| Sao Nicolau | Cape Verde | 2009 | 19 | 1 | 1 | ND |
| Sao Vicente | Cape Verde | 2009 | 19 | 1 | 1 | ND |
| Silhouette | Seychelles | None | NA | 0 | 0 | None found |
| Singapore | Singapore | 1960–2011 | 160, 161, 3 | 53 | 10 | ND |
| Skopelos | Greece | None | NA | 0 | 0 | [European CDC](http://www.ecdc.europa.eu/) |
| Sri Lanka | Sri Lanka | 1965–1966, 1989–2003, 2005–2011 | 162–164 | 22 | 10 | ND |
| St Croix | United States | 1964, 1978, 1981, 1985, 1991, 1993–1996, 2001–2002, 2004, 2009–2010, 2012 | 165–170 | 4 | 5 | ND |
| St Eustatius | Netherlands | 2010–2012 | 4, 147 | 3 | 3 | ND |
| St Kitts | Saint Kitts and Nevis | 1964, 1978, 1981, 1985, 1991, 1993–1996, 2001–2002, 2004–2005, 2009–2010, 2012 | 4, 171–173 | 4 | 5 | ND |
| Subi | Indonesia | None | NA | 0 | 0 | None found |
| Tahaa | French Polynesia | 2001, 2009 | 38, 174 | 1 | 2 | ND |
| Tahiti | French Polynesia | 1944, 1971, 1988–1990, 1996–1997, 2001, 2008–2009, 2013 | 8, 20, 21, 94, 175, 176 | 3 | 5 | ND |
| Taiwan | People's Republic of China | 1931, 1942–1943, 1987–2012 | 177–181 | 23 | 10 | ND |
| Tarawa | Kiribati | 2008 | 183–184 | 1 | 1 | ND |
| Texada | Canada | None | NA | 0 | 0 | [PHA of Canada](http://www.phac-aspc.gc.ca/fluwatch/) |
| Tongatapu | Tonga | 1974–1975, 1980–1983, 1998, 2003–2005, 2007–2009 | 185–189 | 4 | 6 | ND |
| Ua Pou | French Polynesia | 2009 | 187 | 1 | 1 | ND |
| Ulithi | Federated States of Micronesia | 2012 | 190 | 1 | 1 | ND |
| Upolu | Samoa | 1979, 1997, 2008 | 183, 191–194 | 1 | 1 | ND |
| Ustica | Italy | None | NA | 0 | 0 | [European CDC](http://www.ecdc.europa.eu/) |
| Utila | Honduras | 2005 | 195 | 1 | 1 | ND |
| Vanua Levu | Fiji | 1930, 1943, 1989, 1997–1998, 2007–2009, 2011 | 145, 196–201 | 3 | 4 | ND |
| Yap | Federated States of Micronesia | 1988, 1995, 2004, 2011 | 202–205 | 1 | 2 | ND |
| Zanzibar | Tanzania | 2010 | 206 | 1 | 1 | ND |

NA: not available; ND: not done

**References**

1. Horlbeck HB. Dengue. Public Health Papers and Reports 1896;22:191-196.

2. World Health Organization. WHO Report on Global Surveillance of Epidemic-prone Infectious Diseases. <http://www.who.int/csr/resources/publications/surveillance/WHO_CDS_CSR_ISR_2000_1/en/> [accessed 7 August 2013].

3. World Health Organization. DengueNet. <http://apps.who.int/globalatlas/default.asp> [accessed 7 August 2013].

4. World Health Organization. Caribbean Epidemiology Centre Surveillance Report 2012:32(5).

5. Adnkronos International. ProMED. Dengue/DHF Update. 2007 http://www.promedmail.org/direct.php?id=10487 [accessed 7 August 2013].

6. Soedarmo SP. The Epidemiology, Control and Prevention of Dengue Hemorrhagic Fever In Indonesia. Trop. Med. 1993;4:161-172.

7. Yoshikawa MJ, Kusriastuti R. Surge of Dengue Virus Infection and Chikungunya Fever in Bali in 2010: The Burden of Mosquito-Borne Infectious Diseases in a Tourist Destination. Tropical Medicine and Health 2013;41(2):67-78. doi:10.2149/tmh.2011-05.

8. The West Australian. Dengue fever warning for Bali. ProMED. Dengue/DHF Update. 2010 <http://www.promedmail.org/direct.php?id=20101220.4483> [accessed 2 August 2013].

9. Indo Pos Online. ProMED. Dengue/DHF Update. 2008 <http://www.promedmail.org/direct.php?id=20080519.1668> [accessed 2 August 2013].

10. The Jakarta Post. Dengue fever reaches its peak. ProMED. Dengue/DHF Update. 2010 <http://www.promedmail.org/direct.php?id=20100222.0597> [accessed 2 August 2013].

11. Gittens-St. Hilaire M, Clarke-Greenidge N. An analysis of the subtypes of dengue fever infections in Barbados 2003-2007 by reverse transcriptase polymerase chain reaction. Virology Journal 1998;152.

12. World Health Organization. Caribbean Epidemiology Centre Surveillance Report 2008: 28(5).

13. World Health Organization. Caribbean Epidemiology Centre Surveillance Report 2010:30(4).

14. ABS CBN News. Dengue outbreak affects 33 in Biliran. <http://www.abs-cbnnews.com/nation/regions/12/25/08/dengue-outbreak-affects-33-naval-biliran> [accessed 7 February 2013].

15. Public Health Surveillance and Informatics Division, National Epidemiology Center, Department of Health. Philippine Integrated Disease Surveillance and Response: Annual Report 2009. <http://www.uhmis.doh.gov.ph/downloads/14-disease-surveillance/pidsr-annual-report/106-2009-phil-integrated-disease-surveillance-and-response-annual-report.html> [accessed 2 July 2013].

16. Biliran Blogs. Dengue alert up in E. Visayas; cases rising. <http://biliranisland.com/blogs/?p=1966> [accessed 2 July 2013].

17. The Philippine Star. Typhoid fever, dengue cases rising in Eastern Visayas. <http://www.philstar.com/region/660720/typhoid-fever-dengue-cases-rising-eastern-visayas>[accessed 2 July 2013].

18. Sun Star Tacloban Local New. DOH alarmed over rise of dengue cases. <http://www.sunstar.com.ph/tacloban/local-news/2013/07/05/doh-alarmed-over-rise-dengue-cases-290956> [accessed 7 August 2013].

19. US Department of State. Cabo Verde Country Specific Information. <http://travel.state.gov/content/passports/english/country/cabo-verde.html> [accessed 7 August 2013].

20. Deparis X, Chungue E, Pauck S, Roche C, Murgue B, Gleize L. The specific epidemiological surveillance of dengue: the method and its importance since the dengue-2 epidemic in French Polynesia in 1996. Trop. Med. Int. Health 1998;7:566-570.

21. Hubert B, Halstead SB. Dengue 1 virus and dengue hemorrhagic fever, French Polynesia, 2001. Emerg. Infect. Dis. 2009;8:1265-70.

22. Direction de la Sante - Bureau des Pathologies Infectieuses. Surveillance de la dengue en Polynesie francaise - Point epidemiologique mensuel - Situation au 31 octobre 2008. <http://www.spc.int/phs/PPHSN/Surveillance/French-Polynesia/2008/Bulletin_dengue_oct%2008.pdf> [accessed 6 June 2013].

23. Institut de Veille Sanitaire. Bilan epidemiologique Dengue Pacifique Sud. 2012. <http://www.invs.sante.fr/Publications-et-outils/Points-epidemiologiques/Tous-les-numeros/International/Bilan-epidemiologique-Dengue-Pacifique-Sud-Avril-2012> [accessed 5 June 2013].

24. World Health Organization. Weekly epidemiological record. 2009:84;469-476. <http://www.who.int/wer/2009/wer8445.pdf?ua=1> [accessed 6 June 2013].

25. Gregorio-Posadas S, Lofranco V, Auza C, White F, Merin J, Dayrit MM, White M. Dengue Fever Outbreak in Cebu. Phil. Journal of Micro and Inf. Dis. 1989;1:16-20.

26. Mahilum M, Ludwig M, Madon M, Becker N. Evaluation of the present dengue situation and control strategies against Aedes aegypti in Cebu City, Philippines. J Vector Ecol. 2005;2:277-83.

27. Global Nation Inquirer. Dengue cases in Cebu City up 51%. ProMED. Dengue/DHF Update. 2010 <http://www.promedmail.org/direct.php?id=20100720.2435> [accessed 5 June 2013].

28. Sun Star. Dengue drops in Cebu City to 2 cases per 1T person. ProMED. Dengue/DHF Update. 2012 <http://www.promedmail.org/direct.php?id=20120109.1003951> [accessed 5 June 2013].

29. Sun Star. Dengue cases in 2012 double previous year’s due to weather. ProMED. Dengue/DHF Update. 2013 <http://www.promedmail.org/direct.php?id=20130128.1517490> [accessed 5 June 2013].

30. El Mostrador. ProMED. Dengue/DHF Update. 2007 <http://beta.promedmail.org/direct.php?id=20070213.0541> [accessed 4 June 2013].

31. Wilson M, Chen LH. Dengue in the Americas. Dengue Bulletin 26. <http://apps.searo.who.int/PDS_DOCS/B0222.pdf> [accessed 4 June 2013].

32. Canals M, Gonzalez C, Canals A, Figueroa D. Epidemiological dynamics of Dengue on Easter Island. Rev. Chilena Infectol. 2012;4:388-94.

33. Taleo G, Toaliu H, Condon R, Clothier H. Dengue in Port Vila, May-July 1998: A short report demonstrating surveillance in action. Inform’Action 2. <http://www.spc.int/phs/index.php?option=com_docman&task=cat_view&gid=62&Itemid=102> [accessed 4 June 2013].

34. Pacific Islands Report. Eighteen Cases of Dengue Reported in Vanuatu. <http://www.wpro.who.int/southpacific/programmes/communicable_diseases/disease_surveillance_response/PSS-18-April-2014/en/> [accessed 4 June 2013].

35. Arima Y and Matsui T. Epidemiologic update on the dengue situation in the Western Pacific Region, 2010. Western Pacific Surveillance and Response Journal, 2011;2:4-8.

36. World Health Organization. Dengue Outbreak in the Maldives. http://www.searo.who.int/entity/emerging_diseases/links/dengue_outbreak_maldives_2011/en/ [accessed 28 March 2013].

37. Micronesia. ProMED. Dengue/DHF Update. 2012 <http://www.promedmail.org/direct.php?id=20120116.1011474> [accessed 28 March 2013].

38. Hubert B, Halstead SB. Dengue 1 virus and dengue hemorrhagic fever, French Polynesia, 2001. Emerg. Infect. Dis. 2009;8:1265-70.

39. Franco L, Di Caro A, Carletti F, Vapalahti O, Renaudat C, Zeller H, Tenorio A. Recent expansion of dengue virus serotype 3 in West Africa. Euro Surveill. 2010;7.

40. ProMED. Dengue - West Africa. 2010 <http://www.promedmail.org/direct.php?id=20100219.205629> [accessed 3 April 2013].

41. Agencia Angola Press. Government admits Dengue epidemic can become endemic in the archipelago. <http://www.portalangop.co.ao/angola/pt_pt/noticias/africa/2010/0/4/Governo-admite-que-epidemia-Dengue-pode-tornar-endemica-arquipelago,9b9d7087-c110-4234-8be0-4d62a3f25cf8.html> [accessed 3 April 2013].

42. Stout T, Duncan M. War Surgery and Medicine. Wellington: Historical Publications Branch; 1954.

43. Direct Travel Insurance Tuvalu. <https://www.direct-travel.co.uk/tuvalu.aspx?sTab=0#mTab2> [accessed 3 April 2013].

44. Institut de Veille Sanitaire. Dengue Pacifique Sud. ProMED. Dengue/DHF Update. 2007 <http://www.promedmail.org/direct.php?id=20070719.2313> [accessed 14 May 2013].

45. Fauran P, Le Gonidec G, Pujol P. A dengue epidemic in Futuna. Bull Soc Pathol Exot Filiales 1978:6;400-9.

46. Kiedrzynski T, Souares Y, Stewart T. Dengue in the Pacific: an updated story. Pacific Health Dialog 1998;1.

47. Departement International et Tropical. Dengue Pacifique Sud. Institut de Veille Sanitaire. <http://www.invs.sante.fr/presse/2007/le_point_sur/dengue_cas_importes_250107/dengue_cas_importes_250107.pdf> [accessed 14 May 2013].

48. World Health Organization. Wallis and Futuna. Country Health Information Profiles. 2011. <http://www.wpro.who.int/countries/wlf/37WAFpro2011_finaldraft.pdf?ua=1> [accessed 14 May 2013].

49. Ministry of Health. Solomon Islands: Dengue outbreak. 2011 <http://www.arphs.govt.nz/health-information/communicable-disease/dengue-fever-zika-chikungunya#.VNQpcFfF_Pk> [accessed 24 April 2013].

50. The Virgin Islands Daily News. Dengue Fever Hits Turks. <http://news.google.com/newspapers?nid=757&dat=19740405&id=xj1OAAAAIBAJ&sjid=oEYDAAAAIBAJ&pg=2043,3496694> [accessed 24 April 2013].

51. World Health Organization. WHO Report on Global Surveillance of Epidemic-prone Infectious Diseases. <http://www.who.int/csr/resources/publications/surveillance/WHO_CDS_CSR_ISR_2000_1/en/> [accessed 7 August 2013].

52. Tukuitonga CF, Maguire T. An epidemic of type 3 dengue on Niue Island. N Z Med J. 1998:851;500-2.

53. Brown MG, Salas RA, Vickers IE, Heslop OD, Smikle MF. Dengue virus serotypes in Jamaica, 2003-2007. West Indian Med J. 2011:2;114-9.

54. Darcy A, Clothier H, Phillips D, Bakote’e B, Stewart T. Solomon Islands dengue seroprevalence study—previous circulation of dengue confirmed. P N G Med J. 2001:1-2;43-7.

55. ABC Radio Australia. Solomons dengue outbreak claims fourth victim. <http://www.radioaustralia.net.au/international/2013-04-23/solomons-dengue-outbreak-claims-fourth-victim/1120318> [accessed 5 June 2013].

56. Nowell WR. An Annotated Bibliography of the Mosquitoes and Mosquito-borne diseases of Guam. Mosquito Systematics 1976:4;355-385.

57. Winnie W. Control of Dengue/Dengue Haemorrhagic Fever in China. Dengue Bulletin 1997:21;25-30.

58. Li FS, Yang FR, Song JC, Gao H, Tang JQ, Zou CH, Hu BN, Wen SR, Qiu FX. Etiologic and serologic investigations of the 1980 epidemic of dengue fever on Hainan Island, China. Am J Trop Med Hyg. 1986:5;1051-4.

59. Fu-Xi Q, Zhi-guo Z. A Pandemic of Dengue Fever on the Hainan Island. Chin Med J. 1988:7;463-7.

60. Qui FX, Chen QQ, Ho QY, Chen WZ, Zhao ZG, Zhao BW. The first epidemic of dengue hemorrhagic fever in the People’s Republic of China. Am J Trop Med Hyg. 1991:4;364-70.

61. Wu JY, Lun ZR, James AA, Chen XG. Review: Dengue Fever in Mainland China . Am J Trop Med Hyg. 2010:3;664-71.

62. Terra Espana. ProMED. Dengue/DHF Update. 2007 <http://www.promedmail.org/direct.php?id=20070521.1616> [accessed 13 June 2013].

63. ProMED. Dengue - Dominican Republic. 2009 <http://www.promedmail.org/direct.php?id=20091014.3536> [accessed 13 June 2013].

64. La Nacion Dominicana. Medicos especialistas afirman hay brote de dengue en República Dominicana. ProMED. Dengue/DHF Update. 2008 <http://www.promedmail.org/direct.php?id=20081207.3840> [accessed 13 June 2013].

65. El Nacional. Piden por dengue emergencia nacional. ProMED. Dengue/DHF Update. 2010 <http://www.promedmail.org/direct.php?id=20100713.2342> [accessed 13 June 2013].

66. La Hora Nacional. Casos de dengue se triplican en 2012. <http://www.lahora.com.ec/index.php/noticias/show/1101325464/-1/Casos_de_dengue_se_triplican_en_2012.html> [accessed 13 June 2013].

67. Listin Diario. Suman 36 las muertes por dengue. ProMED. Dengue/DHF Update. 2012 <http://www.promedmail.org/direct.php?id=20121125.1423068> [accessed 13 June 2013].

68. El Universo. Quinto muerto por dengue en 55 dias. ProMED. Dengue/DHF Update. 2013 <http://www.promedmail.org/direct.php?id=20130304.1567614> [accessed 13 June 2013].

69. Haveeru. ProMED. Dengue/DHF Update. 2011 <http://www.promedmail.org/direct.php?id=20110705.2035> [accessed 13 June 2013].

70. Rebelo JM, Costa JM, Silva FS, Pereira YN, da Silva JM. Distribution of Aedes aegypti and dengue in the State of Maranhao, Brazil. Cad Saude Publica 1999:3;477-86.

71. Vasconcelos PF, Lima JW, Raposo ML, Rodrigues SG, da Rosa JF, Amorim SM, da Rosa ES, Moura CM, Fonseca N, da Rosa AP. Seroepidemiologic survey in Sao Luis Island, State of Maranhao, Brazil, during a dengue fever epidemics. Rev Soc Bras Med Trop. 1999:2;171-9.

72. Goncalves Neto VS, Rebelo JM. Epidemiological characteristics of dengue in the Municipality of Sao Luis, Maranhao, Brazil, 1997-2002. Cad Saude Publica 2004:5;1424-31.

73. World Health Organization. WHO Report on Global Surveillance of Epidemic-prone Infectious Diseases. <http://www.who.int/csr/resources/publications/surveillance/WHO_CDS_CSR_ISR_2000_1/en/> [accessed 7 August 2013].

74. World Health Organization. Dengue fever in Madeira, Portugal. <http://www.who.int/csr/don/2012_10_17/en/> [accessed 10 August 2013].

75. Castle T, Amador M, Rawlins SJ, Figueroa P, Reiter P. Absence of impact of aerial malathion treatment on Aedes aegypti during a dengue outbreak in Kingston, Jamaica. Rev Panam Salud Publica 1999:2;100-5.

76. Jamaica Observer. Dengue outbreak not over, says health ministry. <http://www.jamaicaobserver.com/latestnews/Dengue-outbreak-not-over--says-health-ministry> [accessed 3 May 2013].

77. Brown MG, Salas RA, Vickers IE, Heslop OD, Smikle MF. Molecular epidemiology of dengue in Jamaica dengue virus genotypes in Jamaica, 2007. West Indian Med J. 2011:2;120-5.

78. World Health Organization. Caribbean Epidemiology Centre Surveillance Report 2002: 22(3).

79. World Health Organization. Caribbean Epidemiology Centre Surveillance Report 2004:3.

80. World Health Organization. Caribbean Epidemiology Centre Surveillance Report 2006:26(3).

81. Taulung L, Asher A. Dengue fever outbreak in Kosrae. InformAction 1998;2:16–8.

82. Metselaar D, Grainger CR, Oei KG, Reynolds DG, Pudney M, Leake CJ, Tukei PM, D’Offay RM, Simpson DIH. An outbreak of type 2 dengue fever in the Seychelles, probably transmitted by Aedes albopictus (Skuse). Bull World Health Organ. 1980:6;937-43.

83. ProMED. Dengue - Seychelles. 2013 <http://www.promedmail.org/direct.php?id=20130212.302675> [accessed 18 June 2013].

84. Fauran P, Moreau JP. Dengue surveillance in New Caledonia and other South Pacific islands. Institut Pasteur. WHO Regional Office for South-East Asia 1984:10;64-69.

85. Institut Pasteur de Nouvelle Caledonie. Laboratory surveillance of dengue fever in New Caledonia - 2004. <https://www.spc.int/phs/PPHSN/Outbreak/Reports/Dengue_fever-surv-NC-2004.pdf> [accessed 3 May 2013].

86. Tropical Medical Bureau/ Report on New Caledonian’s increasing Dengue problem. <http://www.tmb.ie/destinations/news.asp?title=Report-on-New-Caledonians-increasing-Dengue-problem&id=166517> [accessed 24 April 2013].

87. Direction des Affaires Sanitaires et Sociales. Situation Sanitaire en Nouvelle-Caledonie 2012. <http://www.dass.gouv.nc/portal/page/portal/dass/librairie/fichiers/26752273.PDF> [accessed 3 May 2013].

88. European Centre for Disease Prevention and Control. Communicable Disease Threats Report. 2013 <http://www.ecdc.europa.eu/en/publications/Publications/Communicable-disease-threats-report-21-sep-2013.pdf> [accessed 3 May 2013].

89. Alves MJ, Fernandes PL, Amaro F, Osorio H, Luz T, Parreira P, Andrade G, Ze-Ze L, Zeller H. Clinical presentation and laboratory findings for the first autochthonous cases of dengue fever in Madeira island, Portugal, October 2012. Euro Surveill. 2013:6.

90. Pacific Islands Report. 13 Cases of Dengue Reported in Majuro. 2003 <http://pidp.eastwestcenter.org/pireport/2003/October/10-24-03.htm> [accessed 5 June 2013].

91. Radio International New Zealand. CDC official says dengue fever cases in Marshall Islands capital dropping. ProMED. Dengue/DHF Update. 2011 <http://www.promedmail.org/direct.php?id=20111121.3417> [accessed 5 June 2013].

92. Solomon Star. Dengue reported cases now stands at 2226. <http://www.solomonstarnews.com/news/national/17541-dengue-reported-cases-now-stands-at-2226> [accessed 5 June 2013].

93. Pinheiroa FP, Corberb SJ. Global situation of dengue and dengue haemorrhagic fever, and its emergence in the Americas. World Health Stat Q. 1997:3-4;161-9.

94. Chungue E, Deparis X, Bernadette M. Dengue in French Polynesia: Major Features, Surveillance, Molecular Epidemiology and Current Situation. Pacific Health Dialog 1998:1;154-162.

95. Minivan News. ProMED. Dengue/DHF Update. 2008 <http://www.promedmail.org/direct.php?id=20080618.1901> [accessed 9 June 2013].

96. The International Federation of Red Cross and Red Crescent. Maldives Dengue Outbreak. <http://reliefweb.int/sites/reliefweb.int/files/resources/Full_Report_1923.pdf> 2011 [accessed 9 June 2013].

97. Minivan News. Maldives hit by dengue fever in global epidemic. ProMED. Dengue/DHF Update. 2010 <http://www.promedmail.org/direct.php?id=20100727.2520> [accessed 9 June 2013].

98. Minivan News. Maldives grapples with difficult dengue outbreak. <http://minivannews.com/society/maldives-grapples-with-difficult-dengue-outbreak-18733> [accessed 9 June 2013].

99. Minivan News. Construction sites blamed as dengue cases surge. ProMED. Dengue/DHF Update. 2012 <http://www.promedmail.org/direct.php?id=20120326.1080652> [accessed 9 June 2013].

100. Direction de la Sante - Bureau des Pathologies Infectieuses. Bulletin mensuel de surveillance de la dengue en Polynesie Francaise, Decembre 2009. <https://www.spc.int/phs/PPHSN/Surveillance/French-Polynesia/2009/PolynesieF_bulletin_dengue_PF_dec2009.pdf> [accessed 4 June 2013].

101. ProMED. Dengue - Maurice. 2009 <http://www.promedmail.org/direct.php?id=20090617.2234> [accessed 4 June 2013].

102. Global Infectious Diseases and Epidemiology Online Network Database (2011) Dengue - Mayotte. <http://www.gideononline.com/> [accessed 14 May 2013].

103. Sissoko D, Ezzedine K, Giry C, et al. Seroepidemiology of Dengue Virus in Mayotte, Indian Ocean, 2006. Mokrousov I, ed. *PLoS ONE* 2010;5(11):e14141. doi:10.1371/journal.pone.0014141.

104. LINFO. 43 dengue cases and 6 chikungunya cases in Mayotte. ProMED (2012) Dengue/DHF Update. <http://www.promedmail.org/direct.php?id=20120524.1140768> [accessed 14 May 2013].

105. van den Berg H, Velayudhan R, Ebol A, Catbagan BHG, Turingan R, Tuso M, Hii J. Operational efficiency and sustainability of vector control of malaria and dengue: descriptive case studies from the Philippines. Malaria Journal 2012:269.

106. Minda News. Dengue deaths in R-12 now 11. <http://www.mindanews.com/top-stories/2011/08/08/dengue-deaths-in-r-12-now-11/> [accessed 3 April 2013].

107. Minda News. Dengue cases recored in 4 GenSan barangays. ProMED. Dengue/DHF Update. 2012 <http://www.promedmail.org/direct.php?id=20120221.1047463> [accessed 3 April 2013].

108. Effler P, Pang L, Kitsutani P, Vorndam V, Nakata M, Ayers T, Elm J, Tom T, Reiter P, Rigau-Perez JG, Hayes JM, Mills K, Napier M, Clark GG, Gubler DJ. Dengue Fever, Hawaii, 2001 - 2002. Emerg. Infect. Dis. 2005:5;742-749.

109. World Health Organization. WHO Report on Global Surveillance of Epidemic-prone Infectious Diseases. <http://www.who.int/csr/resources/publications/surveillance/WHO_CDS_CSR_ISR_2000_1/en/> [accessed 7 August 2013].

110. World Health Organization. Caribbean Epidemiology Centre Surveillance Report 2003:23(3).

111. Spirit of Montserrat. Health Ministry confirms several dengue cases. <http://zjb.gov.ms/2011/11/03/health-ministry-confirms-several-dengue-cases/> [accessed 3 April 2013].

112. The Montserrat Reporter. Three cases of dengue fever confirmed on Montserrat this week. <http://www.themontserratreporter.com/three-cases-of-dengue-fever-confirmed-on-montserrat-this-week/> [accessed 3 April 2013].

113. Global Infectious Diseases and Epidemiology Online Network Database (2011) Dengue - Moorea-Maiao. <http://www.gideononline.com/> [accessed 9 July 2013].

114. Tahiti News (2013) Dengue: 175 Confirmed Cases. <http://tahitinews.co/english/dengue-175-confirmed-cases/> [accessed 9 July 2013].

115. World Health Organization. Dengue Outbreak in the Maldives. 2011 http://www.searo.who.int/entity/emerging_diseases/links/dengue_outbreak_maldives_2011/en/ [accessed 9 July 2013].

116. Descloux E, Mangeas M, Menkes CE, et al. Climate-Based Models for Understanding and Forecasting Dengue Epidemics. Anyamba A, ed. PLoS Neglected Tropical Diseases 2012;6(2):e1470. doi:10.1371/journal.pntd.0001470.

117. Steel A, Gubler DJ, Bennett SN. Natural attenuation of dengue virus type-2 after a series of island outbreaks: a retrospective phylogenetic study of events in the South Pacific three decades ago. Virology 2010:2;505-12.

118. Global Infectious Diseases and Epidemiology Online Network Database (2011) Dengue - Nukunonu. <http://www.gideononline.com/> [accessed 14 May 2013].

119. Star Advertiser. State receives 12 more suspected cases of dengue. ProMED. Dengue/DHF Update. 2011 <http://www.promedmail.org/direct.php?id=20110404.1041> [accessed 4 June 2013].

120. Sakudo A, Onodera T, Shintani H, Ikuta K Dengue virus presence and surveillance in Okinawa (Review). Exp Ther Med. 2012:1;15-17.

121. Gibbons RV, Streitz M, Babina T, Fried JR. Dengue and US Military Operations from the Spanish-American War through today. Emerg. Infect. Dis. 2012:4;623-30.

122. GMA News. Dengue cases in Western Visayas up 163%. ProMED. Dengue/DHF Update. 2007 <http://www.promedmail.org/direct.php?id=20070821.2726> [accessed 6 March 2013].

123. Angelo FAL (2009) Dengue Rising. <https://scrimgeour.wordpress.com/tag/iloilo-dengue-fever/> [accessed 6 March 2013].

124. The News Today. IPHO verifies ‘dengue outbreak’ in Tubungan town. ProMED. Dengue/DHF Update. 2010 <http://www.promedmail.org/direct.php?id=20100601.1821> [accessed 6 March 2013].

125. Sun Star. Mayor: rise in dengue cases alarming. ProMED. Dengue/DHF Update. 2012 <http://www.promedmail.org/direct.php?id=20120617.1170689> [accessed 6 March 2013].

126. Panay News. Iliolo City dengue cases down but… <http://panaynewsphilippines.com/2014/05/23/iloilo-city-dengue-cases-down-but/> [accessed 28 May 2014].

127. Ashford DA, Savage HM, Hajjeh RA, Mcready J, Bartholomew DM, Spiegel RA, Vorndam V, Clark GG, Gubler DG. Outbreak of Dengue Fever in Palau, Western Pacific: Risk Factors For Infection. Am J Trop Med Hyg. 2003:2;135-40.

128. Phuket Gazette. Fears rise over potential dengue outbreak.. <http://www.healthmap.org/admin/disp.php?id=22923> [accessed 2 May 2013].

129. Phuket Wan. Phuket D-Day as Dengue Fever Threat Skyrockets. <http://phuketwan.com/tourism/phuket-day-dengue-threat-increases-12614/> [accessed 2 May 2013].

130. Phuket News. 123 dengue fever patients found in Phuket. ProMED. Dengue/DHF Update. 2010 <http://www.promedmail.org/direct.php?id=20100510.1528> [accessed 4 June 2013].

131. The Phuket News. Phuket dengue fever cases on the rise. ProMED. Dengue/DHF Update. 2012 <http://www.promedmail.org/direct.php?id=20120603.1154583> [accessed 2 May 2013].

132. Phuket News. Phuket reports 323 cases of dengue fever. <http://phuketnews.phuketindex.com/features/phuket-reports-323-cases-of-dengue-fever-196436.html> [accessed 2 May 2013].

133. Global Infectious Diseases and Epidemiology Online Network Database (2011) Dengue - Pinang. <http://www.gideononline.com/> [accessed 14 May 2013].

134. ProMED. Dengue - Malaysia. 1995 <http://www.promedmail.org/direct.php?id=19950824.0744> [accessed 14 May 2013].

135. People’s Daily Online. Dengue fever claims 40 lives in MalaysiaProMED. Dengue/DHF Update. 2009 <http://www.promedmail.org/direct.php?id=20090428.1595> [accessed 14 May 2013].

136. Shepard DS, Lees R, Ng CW, Undurraga EA, Halasa Y, Lum L. Burden of Dengue in Malaysia. Report from a Collaboration between Universities and the Ministry of Health of Malaysia. Waltham, MA: Brandeis University, Schneider Institutes for Health Policy; 2012.

137. The Star. Dengue cases in Penang drop by a third. <http://thestar.com.my/metro/story.asp?file=/2011/12/9/north/10061759&sec=north> [accessed 14 May 2013].

138. The Borneo Post. Number of dengue cases drops in 10^th^ week of this year.. <http://www.theborneopost.com/2012/03/16/number-of-dengue-cases-drops-in-10th-week-of-this-year/> [accessed 14 May 2013].

139. Robertson AS, Upson D, Dever G, Tipene-Leach D. Community-based research and medical education: the PBMOTP experience with dengue, leptospirosis, TB and intestinal parasites. Pacific Health Dialog 1996:2;178-86.

140. Radio New Zealand International. 460 people in Cook Islands affected by Dengue Fever outbreak. ProMED. Dengue/DHF Update. 2007 <http://www.promedmail.org/direct.php?id=20070121.0284> [accessed 14 May 2013].

141. Radio New Zealand International. Dengue outbreak in Cooks affects more than 60 people. ProMED. Dengue/DHF Update. 2009 <http://www.promedmail.org/direct.php?id=20090413.1412> [accessed 14 May 2013].

142. D’Ortenzio E, Balleydier E, Baville M, Filleul L, Renault P. Dengue fever in the Reunion Island and in South Western islands of the Indian Ocean. Medicine et Maladies Infectieuses 2011:9;475-9.

143. Larrieu S, Dehecq JS, Balleydier E, Jaffar MC, Michault A, Vilain P, Leparc-Goffart I, Polycarpe D, Filleul L. Re-emergence of dengue in Reunion, France, January to April 2012. Euro Surveill. 2012:20.

144. Figueroa M, Pereira R, Gutierrez H, de Mejia C, Padilla N. LA EPIDEMIA DE DENGUE EN HONDURAS, 1978-1980. Boletin de la Oficina Sanitaria Panamericana 1982:5; 434-41.

145. Maguire T, Miles JAR, Macnamara FN, Wilkinson PJ, Austin FJ, Mataika JU. Mosquito-borne infections in Fiji: V. The 1971-73 dengue epidemic. *The Journal of Hygiene* 1974;2:263-270.

146. Centers for Disease Control and Prevention. Outbreak Notice Update: Dengue, Tropical and Subtropical Regions. <http://nontradmd.blogspot.com/2008/11/cdc-on-dengue.html> [accessed 20 February 2013].

147. Pan American Health Organization. Netherland Antilles. Health in the Americas. <http://www.paho.org/saludenlasamericas/index.php?option=com_docman&task=doc_view&gid=139&Itemid>= 2012 [accessed 20 February 2013].

148. Lambrechts L, Scott TW, Gubler DJ. Consequences of the Expanding Global Distribution of Aedes albopictus for Dengue Virus Transmission. Halstead SB, ed. PLoS Neglected Tropical Diseases 2010;4(5):e646. doi:10.1371/journal.pntd.0000646.

149. World Health Organization. Dengue fever in Cape Verde - update 1. http://www.who.int/csr/don/2009_11_18/en/ [accessed 25 April 2013].

150. Philippine Star. Dengue outbreak declared in 8 N. Samar Villages. <http://www.philstar.com/nation/9917/dengue-outbreak-declared-8-n-samar-villages> [accessed 25 April 2013].

151. Samar News. DOH: Dengue is more deadly than AH1N1. <http://www.samarnews.com/news2009/jun/f2264.htm> [accessed 25 April 2013].

152. Public Health Surveillance and Informatics Division, National Epidemiology Center, Department of Health. Disease Surveillance Report: Morbidity Week 4^th^ - January 1 - 30, 2009. <http://www.doh.gov.ph/sites/default/files/2009Den04WMR.pdf> [accessed 26 April 2013].

153. Samar News. East Samar creates Outbreak Response Team vs. dengue. <http://www.samarnews.com/news2010/jul/a399.htm> [accessed 26 April 2013].

154. Philippine Information Agency. Samar observes ASEAN Dengue Day. <http://news.pia.gov.ph/archives/cy2012/18375> 2012 [accessed 26 April 2013].

155. Sun Star Tacloban Local News. Health department: Dengue fever cases in Eastern Visayas declining. <http://www.sunstar.com.ph/tacloban/local-news/2011/08/11/health-department-dengue-fever-cases-eastern-visayas-declining-172376> [accessed 25 April 2013].

156. Sun Star Tacloban Local News. Health department to curb spread of chikungunya. <http://www.sunstar.com.ph/tacloban/local-news/2013/01/29/health-department-curb-spread-chikungunya-265439> [accessed 25 April 2013].

157. Sun Star Tacloban Local News. DOH reminds public on dengue, chikungunya. <http://www.sunstar.com.ph/tacloban/local-news/2013/01/21/doh-reminds-public-dengue-chikungunya-263978> [accessed 25 April 2013].

158. European Centre for Disease Prevention and Control. Dengue in the Galapagos islands, Ecuador. 2010 <http://www.ecdc.europa.eu/en/activities/sciadvice/_layouts/forms/Review_DispForm.aspx?ID=290&List=a3216f4c-f040-4f51-9f77-a96046dbfd72> [accessed 26 April 2013].

159. Maria de Lourdes Monteiro. Surveillance Service of the Ministry of Health. ProMED. Dengue/DHF Update. 2010 <http://www.promedmail.org/direct.php?id=20100913.3308> [accessed 3 April 2013].

160. Shepard DS, Undurraga EA, Halasa YA. Economic and Disease Burden of Dengue in Southeast Asia. Gubler DJ, ed. PLoS

Neglected Tropical Diseases2013;7(2):e2055. doi:10.1371/journal.pntd.0002055.

161. Yew YW, Ye T, Ang LW, Ng LC, Yap G, James L, Chew SK, Goh KT. Seroepidemiology of Dengue Virus Infection Among Adults in Singapore. Ann Acad Med Singapore 2009:8;667-75.

162. Messer WB, Vitaran UT, Sivananthan K, Elvtigala J, Preethimala LD, Ramesh R, Withana N, Gubler DJ, De Silva AM. Epidemiology of dengue in Sri Lanka before and after the emergence of epidemic dengue hemorrhagic fever. Am J Trop Med Hyg. 2002:6;765-73.

163. Kanakaratne N, Wahala M, Wahala PB, Messer WB, Tissera HA, Shahani A, Abeysinghe N, de Silva AM, Gunasekera M. Severe Dengue Epidemics in Sri Lanka, 2003-2006. Emerg. Infect. Dis. 2009:2;192-199.

164. Epidemiology Unit Ministry of Health Sri Lanka. National Plan of Action for Prevention and Control of Dengue Fever 2005 - 2009. <http://www.epid.gov.lk/web/images/pdf/Circulars/latest_draft_poa_for_dfdhf.pdf> [accessed 8 August 2013].

165. Gubler DJ, Vorndam V, Clark GG. Dengue Surveillance—United States, 1986-1992. Morbidity and Mortality Weekly Report 1994: SS-2;7-19.

166. Mohammed H, Ramos M, Armstrong J, et al. An Outbreak of Dengue Fever in St. Croix (US Virgin Islands), 2005. Myer L, ed. *PLoS ONE* 2010;5(10):e13729. doi:10.1371/journal.pone.0013729.

167. United States Virgin Islands Department of Health. Health Officials: CDC Working with Department of Health to Investigate Dengue in St. Croix. <http://www.healthvi.org/news/press-releases/2012/12/cdc-working-with-department-of-health-to-investigate-dengue.html> [accessed 25 April 2013].

168. Morris T, Lang F, Christopher R, Plaskett D, Biggerstaff B, Horiuchi K, Han G, Ellis B, Amador M, Felix G, Beltran M, Tomashek K, Munoz-Jordan J, Hunsperger E, Barrera R, Margolis H, Thomas D, Ellis E. School Reporting of a Dengue Outbreak - St. Croix, U.S. Virgin Islands, 2012. Morbidity and Mortality Weekly Report 2013:9;172.

169. St. Croix Source. Dengue Still a Problem, Health Commissioner Tells Senate Committee. <http://stcroixsource.com/content/news/local-news/2013/02/21/dengue-still-problem-health-commissioner-tells-senate-committeee> [accessed 7 March 2013].

170. St. Croix Source. Dengue Fever Cases Continue into November. <http://stcroixsource.com/content/news/local-news/2012/11/14/dengue-fever-cases-continue-november> [accessed 7 March 2013].

171. World Health Organization. WHO Report on Global Surveillance of Epidemic-prone Infectious Diseases. <http://www.who.int/csr/resources/publications/surveillance/WHO_CDS_CSR_ISR_2000_1/en/> [accessed 7 August 2013].

172. Pan American Health Organization. Number of Reported Cases of Dengue and Severe Dengue (SD) in the Americas, by Country: Figures for 2010 (to week noted by each country). <http://new.paho.org/hq/dmdocuments/2010/dengue_cases_2010_december_10_2%20.pdf> [accessed 7 August 2013].

173. Flu Trackers. Eight Confirmed Dengue Cases In St. Kitts. <https://flutrackers.com/forum/forum/emerging-diseases-other-health-threats-alphabetical-a-thru-h/dengue/94240-st-kitts-and-nevis-dengue-cases> [accessed 8 August 2013].

174. Direction de la Sante - Bureau des Pathologies Infectieuses. Surveillance de la dengue en Polynesie francaise - Bulletin de la semaine 12. 2009 <http://www.hygiene-publique.gov.pf/IMG/pdf/bulletin_surv_pf_sem_12-2014.pdf> [accessed 24 April 2013].

175. Cao-Lormeau V-M, Roche C, Aubry M, et al. Recent Emergence of Dengue Virus Serotype 4 in French Polynesia Results from Multiple Introductions from Other South Pacific Islands. Ooi EE, ed. *PLoS ONE* 2011;6(12):e29555. doi:10.1371/journal.pone.0029555.

176. Tahiti Press. Dengue 4 stabilisation but the epidemic is not over. ProMED. Dengue/DHF Update. 2009 http://www.promedmail.org/direct.php?id=313058 [accessed 3 April 2013].

177. Chang SF, Huang JH, Chen LK, Su CL, Liao TL, Chien LJ, Lin TH, Su CJ, Shu PY. Retrospective serological study on sequential dengue virus serotypes 1 to 4 epidemics in Tainan City, Taiwan, 1994 to 2000. J Microbiol Immunol Infect. 2008:5;377-85.

178. Chen WC, King CC, Chien LY, Chen Sl, Fang AH. Changing prevalence of antibody to Dengue virus in paired sera in the two years following an epidemic in Taiwan. Epidemiol Infect. 1997:2;277-9.

179. Lin CH, Schioler KL, Jepsen MR, Ho CK, Li SH, Konradsen F. Dengue Outbreaks in High-Income Area, Kaohsiung City, Taiwan, 2003-2009. Emerg. Infect. Dis. 2012:10;1603-11.

180. Epoch Times. Home garden landscaping mosquito breeding ground. <http://www.healthmap.org/a.php?555602&trto=en&trfr=zh&pid3390> [accessed 24 April 2013].

181. Taiwan Headlines. ProMED. Dengue/DHF Update. 2011 <http://www.promedmail.org/direct.php?id=20110905.2707> [accessed 24 April 2013].

182. Focus Taiwan News. Dengue fever case confirmed in Kaohsiung: CDC. ProMED. Dengue/DHF Update. 2012 <http://www.promedmail.org/direct.php?id=20120528.1147589> [accessed 24 April 2013].

183. Li D, Liu W, Guigon A, Mostyn C, Grant R, Aaskov J. Rapid Displacement of Dengue Virus Type 1 by Type 4, Pacific Region, 2007-2009. Emerg. Infect. Dis. 2010:1;123-5.

184. Flu Trackers. Dengue Outbreak Confirmed On Tarawa. 2008 <https://flutrackers.com/forum/forum/emerging-diseases-other-health-threats-alphabetical-a-thru-h/dengue/34028-dengue-outbreak-confimed-on-tarawa-kiribati> [accessed 24 April 2013].

185. Gubler DJ, Reed D, Rosen L, Hitchcock Jr JC. Epidemiologic, clinical, and virologic observations on dengue in the Kingdom of Tonga. Am J Trop Med Hyg 1978:3;581-9.

186. Global Infectious Diseases and Epidemiology Online Network Database (2011) Dengue - Tongatapu. <http://www.gideononline.com/> [accessed 14 May 2013].

187. La Depeche. 167 cases of dengue type 4. <http://www.ladepeche.pf/article/social/167-cas-de-dengue-de-type-4> [accessed 24 April 2013].

188. Tonga Review. ProMED. Dengue/DHF Update. 2008 <http://www.promedmail.org/direct.php?id=20080309.0962> [accessed 14 May 2013].

189. Berger S. Infectious Diseases of Tonga. Los Angeles, California: GIDEON Informatics; 2014:80.

190. Micronesia. ProMED. Dengue/DHF Update. 2012 <http://www.promedmail.org/direct.php?id=20120116.1011474> [accessed 4 June 2013].

191. ProMED. Dengue - Samoa and Fiji. 1997 <http://www.promedmail.org/direct.php?id=19971003.2085> [accessed 11 March 2013].

192. ProMED. Samoa. 1997 <http://www.promedmail.org/direct.php?id=19971119.2326> [accessed 11 March 2013].

193. The Fiji Times. Pacific hit by dengue pandemic. <http://www.fijitimes.com/story.aspx?id=103700> [accessed 11 March 2013].

194. Samoa News. Samoa dengue alert has DoH ‘monitoring’ ports. <http://www.samoanews.com/content/samoa-dengue-fever-alert-has-doh-%E2%80%98monitoring%E2%80%99-ports> [accessed 11 March 2013].

195. Utila East Wind. Dengue Sweeps Island. <http://www.aboututila.com/Utila-East-Wind/July-2005/All%20Pages.pdf> [accessed 8 March 2013].

196. Fagbami AH, Mataika PJU, Shrestha M, Gubler DJ. Dengue type 1 epidemic with haemorrhagic manifestations in Fiji, 1989-90. Bull World Health Organ. 1995:3;291-7.

197. ProMED. Dengue - Fiji. 2008 <http://www.promedmail.org/direct.php?id=2193335> [accessed 12 March 2013].

198. Radio New Zealand International. More typhoid and dengue cases reported in Fiji. <http://www.rnzi.com/pages/news.php?op=read&id=31587> [accessed 12 March 2013].

199. The Fiji Times. 11 North dengue cases. <http://www.fijitimes.com/story.aspx?ref=archive&id=100790> [accessed 12 March 2013].

200. The Fiji Times. Hospitals on dengue alert. <http://www.fijitimes.com/story.aspx?id=112324> [accessed 12 March 2013].

201. Fiji. ProMED (2011) Dengue/DHF Update. <http://beta.promedmail.org/direct.php?id=20110606.1725> [accessed 12 March 2013].

202. Singh N, Kiedrzynski T, Lepers C, Benyon EK. Dengue in the Pacific - an update of the current situation. Pacific Health Surveillance and Response 2005:2;111-9.

203. Savage HM, Fritz CL, Rutstein D, Yolwa A, Vorndam V, Gubler DJ. Epidemic of dengue-4 virus in Yap State, Federated States of Micronesia, and implication of Aedes hensilli as an epidemic vector. The Am J Trop Med Hyg 1998:4;519-24.

204. Durand MA, Bel AM, Ruwey I, Marfel M, Yug L, Ngaden V. An outbreak of dengue fever in Yap State. Pacific Health Dialog 2005;2:99-102.

205. ABC Radio Australia. Yap dengue virus differs from Marshall Islands outbreak. <http://www.radioaustralia.net.au/international/radio/onairhighlights/yap-dengue-virus-differs-from-marshall-islands-outbreak> [accessed 8 March 2013].

206. Gautret P, Simon F, Askling HH, Bouchaud O, Leparc-Goffart I, Ninove L, Parola P. Dengue type 3 virus infections in European travellers returning from the Comoros and Zanzibar, February - April 2010. Euro Surveill. 2008;15.
